# Brainstem encoding of speech and musical stimuli in congenital amusia: evidence from Cantonese speakers

**DOI:** 10.3389/fnhum.2014.01029

**Published:** 2015-01-06

**Authors:** Fang Liu, Akshay R. Maggu, Joseph C. Y. Lau, Patrick C. M. Wong

**Affiliations:** ^1^Department of Linguistics and Modern Languages, The Chinese University of Hong KongHong Kong, China; ^2^The Chinese University of Hong Kong – Utrecht University Joint Center for Language, Mind and BrainHong Kong, China; ^3^Roxelyn and Richard Pepper Department of Communication Sciences and Disorders, Northwestern UniversityEvanston, IL, USA; ^4^Department of Otolaryngology, Head and Neck Surgery, Northwestern University Feinberg School of MedicineChicago, IL, USA

**Keywords:** congenital amusia, brainstem, pitch, lexical/musical tone, speech in noise, Cantonese, frequency-following response (FFR)

## Abstract

Congenital amusia is a neurodevelopmental disorder of musical processing that also impacts subtle aspects of speech processing. It remains debated at what stage(s) of auditory processing deficits in amusia arise. In this study, we investigated whether amusia originates from impaired subcortical encoding of speech (in quiet and noise) and musical sounds in the brainstem. Fourteen Cantonese-speaking amusics and 14 matched controls passively listened to six Cantonese lexical tones in quiet, two Cantonese tones in noise (signal-to-noise ratios at 0 and 20 dB), and two cello tones in quiet while their frequency-following responses (FFRs) to these tones were recorded. All participants also completed a behavioral lexical tone identification task. The results indicated normal brainstem encoding of pitch in speech (in quiet and noise) and musical stimuli in amusics relative to controls, as measured by FFR pitch strength, pitch error, and stimulus-to-response correlation. There was also no group difference in neural conduction time or FFR amplitudes. Both groups demonstrated better FFRs to speech (in quiet and noise) than to musical stimuli. However, a significant group difference was observed for tone identification, with amusics showing significantly lower accuracy than controls. Analysis of the tone confusion matrices suggested that amusics were more likely than controls to confuse between tones that shared similar acoustic features. Interestingly, this deficit in lexical tone identification was not coupled with brainstem abnormality for either speech or musical stimuli. Together, our results suggest that the amusic brainstem is not functioning abnormally, although higher-order linguistic pitch processing is impaired in amusia. This finding has significant implications for theories of central auditory processing, requiring further investigations into how different stages of auditory processing interact in the human brain.

## INTRODUCTION

Congenital amusia is a neuro-genetic disorder of musical processing (Drayna et al., 2001; Peretz et al., 2007), affecting around 4% of the general population for both tone and non-tonal language speakers (Kalmus and Fry, 1980; Nan et al., 2010; Wong et al., 2012; although see Henry and McAuley, 2010, 2013, for criticisms). Impacting basic music production and perception abilities (Ayotte et al., 2002), this disorder is also associated with impaired fine-grained pitch discrimination (Peretz et al., 2002; Jiang et al., 2011), elevated thresholds for pitch change detection and pitch direction identification/discrimination (Foxton et al., 2004; Hyde and Peretz, 2004; Liu et al., 2010, 2012b; Jiang et al., 2013), and impaired short-term memory for pitch (Gosselin et al., 2009; Tillmann et al., 2009; Williamson and Stewart, 2010; Albouy et al., 2013a).

Although some early studies reported ceiling performance of amusics on speech intonation processing, presumably due to the coarse intonational contrasts used (Ayotte et al., 2002; Peretz et al., 2002; Patel et al., 2005), more recent research has suggested that amusia is a domain-general pitch processing deficit that also compromises subtle aspects of pitch processing in speech, including lexical tone perception, linguistic and emotional prosody processing, and speech intonation imitation (Patel et al., 2008; Hutchins et al., 2010; Jiang et al., 2010, 2012a,b; Liu et al., 2010, 2012a, 2013; Nan et al., 2010; Tillmann et al., 2011a,b; Thompson et al., 2012). The non-modularity of pitch deficits in amusia has recently been confirmed by a quantitative review through meta-analysis of the previous studies (Vuvan et al., 2014). Furthermore, amusics also show impaired time matching abilities in speech imitation (Liu et al., 2013), and demonstrate reduced speech comprehension in both quiet and noise, with either natural or flattened pitch contours (Liu et al., 2015).

Structural neuroimaging studies suggest that the amusic brain differs from neurotypical brains in subtle ways. For example, the amusic brain has reduced white matter and increased gray matter in the right inferior frontal gyrus (Hyde et al., 2006; Albouy et al., 2013a), reduced gray matter in the right superior temporal gyrus (Albouy et al., 2013a), and thicker cortex in the right inferior frontal gyrus and the right superior temporal gyrus (Hyde et al., 2007). Furthermore, amusics also show reduced gray matter in the left inferior frontal gyrus and the left superior temporal sulcus (Mandell et al., 2007), and reduced arcuate fasciculus connectivity along the right frontotemporal pathway (Loui et al., 2009).

Despite anatomical abnormalities of the amusic brain, most event-related potentials (ERPs) studies have revealed near-normal early pre-attentive brain potentials of amusics (e.g., N100, N200, MMN, P200) in response to small pitch changes and musical incongruities/violations (Peretz et al., 2005, 2009; Moreau et al., 2009, 2013; Mignault Goulet et al., 2012; Omigie et al., 2013), which they often fail to detect at the behavioral level, nor do they respond through late brain potentials that represent attentive processing (e.g., P300, P600; Peretz et al., 2009; Jiang et al., 2012a; Mignault Goulet et al., 2012; Moreau et al., 2013). Furthermore, in an functional magnetic resonance imaging (fMRI) study, amusics demonstrated normal brain activities in both the left and right auditory cortices when passively listening to pure-tone sequences with varying pitch distances, although abnormal deactivation in the right inferior frontal gyrus, decreased connectivity along the right frontotemporal pathway, and abnormal over-connectivity between the left and right auditory cortices were also observed (Hyde et al., 2011). Together, these findings suggest that amusics lack the ability to process subtle pitch variations and music structure consciously, albeit they can do so pre-attentively. Therefore, it seems that the amusic deficits are outside of the auditory cortex, which is assumed to generate early brain potentials, but instead reside in the right inferior frontal gyrus and the right frontotemporal pathway (Hyde et al., 2011; Peretz, 2013).

Nevertheless, a recent magnetoencephalography (MEG) study revealed a decreased and delayed N100m in bilateral inferior frontal gyrus and Heschl’s gyrus/superior temporal gyrus of the amusic brain during a melodic contour discrimination task, suggesting that pitch processing deficits in amusia might start from the auditory cortex (Albouy et al., 2013a). This is consistent with a few other ERP studies which have also observed abnormal early brain responses (e.g., N100, MMN) in amusia (Braun et al., 2008; Jiang et al., 2012a; Omigie et al., 2013). Thus, it remains to be determined at what stage(s) of auditory processing deficits in amusia arise.

At the behavioral level, amusics also demonstrate preserved pitch and music processing abilities in an implicit manner, but not explicitly like typical listeners do. For example, amusics were better able to imitate than identify/discriminate pitch direction and speech intonation, presumably because of unconscious pitch processing during imitation (Loui et al., 2008; Liu et al., 2010; Hutchins and Peretz, 2012). In a melodic discrimination task, amusics showed implicit processing of melodic structure in Western tonal music through faster responses times (but not better performance) to tonal than atonal sequences (Albouy et al., 2013b). They also exhibited implicit processing of melodic expectation (high versus low probability notes) and harmonic structure in priming tasks (Omigie et al., 2012; Tillmann et al., 2012), but were unable to perform as well as controls in the explicit rating task (Omigie et al., 2012). Similarly, although amusics self-reported to be unable to recognize melodies without lyrics, they rated familiarity of instrumental music as well as controls, demonstrating implicit storage of familiar melodies in long-term memory (Tillmann et al., 2014).

The domain-generality and the neural origin(s) of the amusic deficits, together with the intriguing dissociation between pre-attentive/implicit and attentive/explicit processing of pitch in amusia, warrant further investigation. In particular, it is unclear whether the cortical dysfunctions revealed by previous studies were driven by the ascending pathway that started earlier, e.g., the brainstem, and if so, whether the domain-generality of pitch processing also manifests at the brainstem level in amusia. It has been shown that the ability to decode speech/music sounds in a meaningful manner is a complex task involving multiple stages of neural processing (Poeppel and Hickok, 2004; Peretz and Zatorre, 2005; Stewart et al., 2006; Hickok and Poeppel, 2007; Poeppel et al., 2008). Before speech/music sounds can be perceived and mapped onto long-term mental representations in the cortex, relevant acoustic properties such as temporal and spectral information must be represented and transformed through a neural code by subcortical structures, including the auditory nerve, the brainstem, the midbrain, etc. (Eggermont, 2001). It is well established that the auditory brainstem represents elements of speech/music sounds, such as timing, frequency, and timbre, with remarkable fidelity (Chandrasekaran and Kraus, 2010; Skoe and Kraus, 2010). This representation is also influenced by language experience (Krishnan et al., 2005, 2010b,c; Krizman et al., 2012), musicianship (Musacchia et al., 2007; Wong et al., 2007; Parbery-Clark et al., 2009a; Strait et al., 2012), and short-term auditory training (Song et al., 2008; Carcagno and Plack, 2011; Chandrasekaran et al., 2012; Anderson et al., 2013). Given the impact of long-term experience on the brainstem, it is necessary to examine whether amusics’ deficits start earlier at the brainstem, rather than being confined within the cortex, for both musical and speech stimuli.

In addition, given that musicians demonstrate enhanced brainstem encoding of speech and music stimuli as well as speech in noise compared to non-musicians (Musacchia et al., 2007; Wong et al., 2007; Parbery-Clark et al., 2009a; Strait et al., 2012), it will be interesting to examine whether amusics would show reduced brainstem encoding of these stimuli relative to controls. The answer to this question would help establish whether subcortical encoding of speech/music sounds reflects musical aptitude along the entire spectrum from musicians to non-musician controls and to amusics.

In order to most comprehensively interrogate whether pitch-processing deficits in amusia originate from the brainstem, we examined amusics’ frequency-following responses (FFRs) to an array of stimuli in the present study, including speech-in-quiet, speech-in-noise, and musical tones. FFR is scalp-recorded electrophysiological response originated from the rostral brainstem, reflecting phase-locked neural activity in the brainstem (Smith et al., 1975; Sohmer et al., 1977). It is synchronized to the temporal structure (and thus periodicity) of the evoking stimulus (Chandrasekaran and Kraus, 2010; Skoe and Kraus, 2010). Thus, FFR has the potential to reveal subtle pitch tracking problems of amusics that cortical ERPs cannot. Apart from FFR recordings, we also examined whether there is a dissociation between brainstem representation and behavioral identification of pitch in amusia.

**FIGURE 1:**
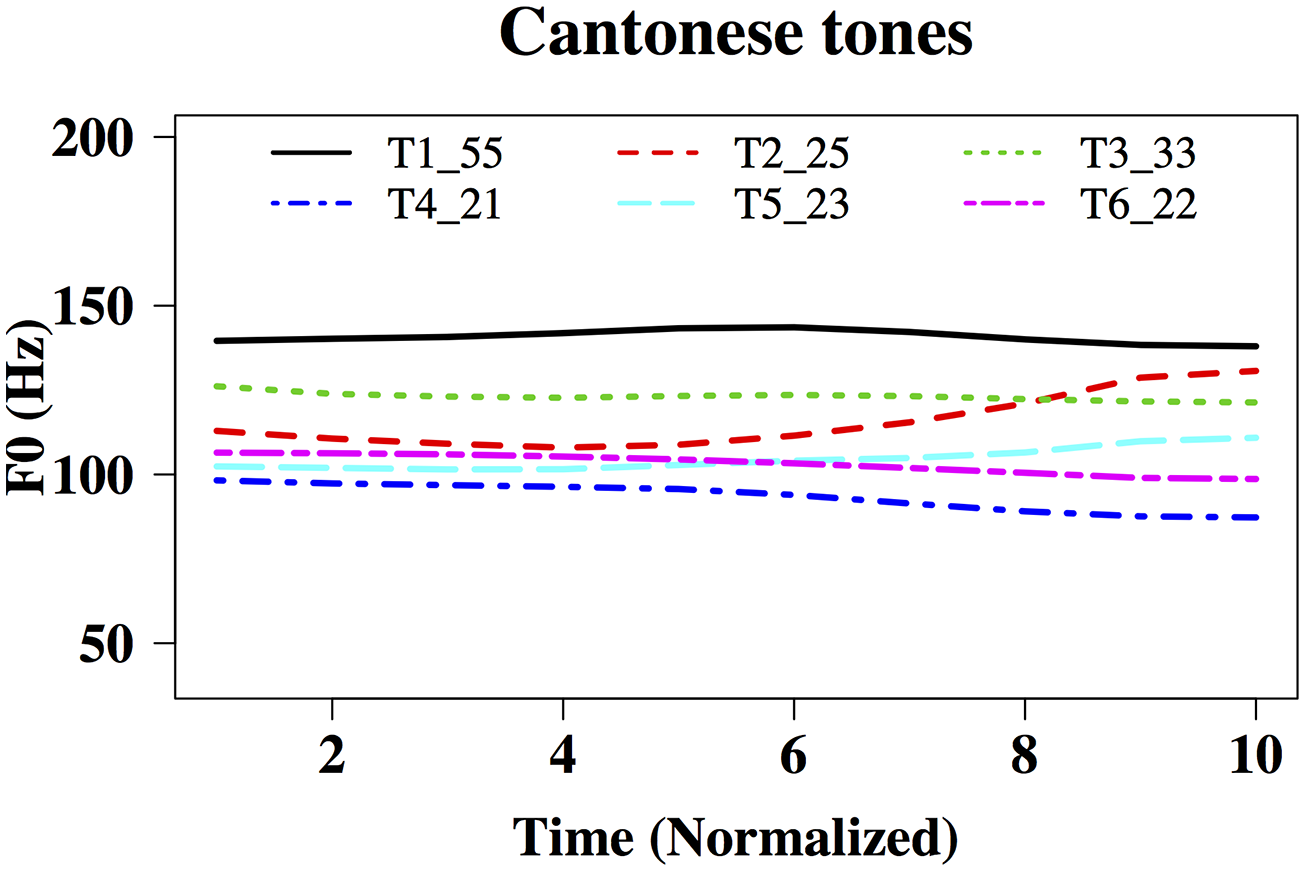
**Time-normalized F0 contours of the six Hong Kong Cantonese tones on the syllable /ji/** T1: high-level, 55, 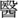, ‘doctor’; T2: high-rising, 25, 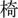, ‘chair’; T3: mid-level, 33, 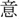, ‘meaning’; 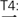 low-falling, 21, 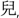, ‘son’; T5: mid-rising, 23, 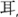, ‘ear’; T6: low-level, 22, 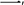, ‘two.’ F0 ranges: 138–145 Hz, 107–140 Hz, 121–128 Hz, 87–99 Hz, 100–115 Hz, and 96–106 Hz, respectively.

In order to elicit meaningful identification of natural and behaviorally relevant pitches, we used native speakers of Cantonese as participants and Cantonese tones as part of test materials. Cantonese is a tone language with a highly complex tonal system (Yip, 2002). There are six lexical tones in Cantonese, named Tone 1 to Tone 6. Using the scale of 1–5 (5 being the highest and 1 being the lowest tonal point; Chao, 1930), the high-level Cantonese Tone 1 can be transcribed as having pitch values of 55, the high-rising Tone 2 as having 25, the mid-level Tone 3 as 33, the low-falling Tone 4 as 21, the low-rising Tone 5 as 23, and the low-level Tone 6 as 22 (**Figure 1**). The fact that there are multiple tones with similar acoustic features in Cantonese (Rising: 25/23; Level: 55/33/22; Falling: 21) makes tone perception challenging even for native speakers (Varley and So, 1995; Ciocca and Lui, 2003; Francis and Ciocca, 2003). It has been found that tone language speakers have enhanced brainstem encoding of native tonal features, e.g., acceleration rates of pitch rises (Krishnan et al., 2005, 2010b,c), presumably because of perceptual learning of native sounds through long-term exposure to the sound environment. Whether amusic tone language speakers would demonstrate such a brainstem-encoding enhancement like typical listeners do is an open question. Given that lexical and non-lexical pitch processing across different tone language speakers (e.g., Chinese versus Thai) differentiate themselves cortically (Gandour et al., 2002), but not subcortically (Krishnan et al., 2010b), examining tone language speakers and lexical tone processing would enable us to determine whether or not there is a dissociation between brainstem representation and behavioral identification of pitch in amusia.

To this end, we conducted a set of FFR experiments to examine whether Cantonese-speaking amusics have deficits in brainstem encoding of speech (in quiet and noise) and musical stimuli (in quiet). During the experiments, participants listened passively to lexical and musical tones while their brain activities were recorded. From the FFR waveforms, we measured the temporal precision, neural tuning/phase-locking, and amplitude of the synchronous neural responses with respect to the periodicity of the stimuli. Apart from FFR recordings, all participants also completed a behavioral task, in which they were required to identify the words that corresponded to the six Cantonese tones. Identification accuracy and response times were calculated.

## MATERIALS AND METHODS

### PARTICIPANTS

Participants (*n* = 14 in each group) were recruited by advertisements through mass mail services at the Chinese University of Hong Kong. All participants had normal hearing in both ears, with pure-tone air conduction thresholds of 25 dB HL or better at frequencies of 0.5, 1, 2, and 4 kHz. All were native speakers of Hong Kong Cantonese, and none reported having speech or hearing disorders or neurological/psychiatric impairments in the questionnaires regarding their music, language, and medical background. Written informed consents were obtained from all participants prior to the experiments. The Institutional Review Board of Northwestern University and The Joint Chinese University of Hong Kong – New Territories East Cluster Clinical Research Ethics Committee approved the study.

All participants were right-handed as assessed by the Edinburgh Handedness Inventory (Oldfield, 1971). The diagnosis of amusia in these participants was conducted using the Montreal Battery of Evaluation of Amusia (MBEA; Peretz et al., 2003), which consists of six subtests measuring the perception of scale, contour, interval, rhythm, and meter of Western melodies, and recognition memory of these melodies. Those who scored 65 or under on the pitch composite score (sum of the scores on the scale, contour, and interval subtests) or below 78% correct on the MBEA global score were classified as amusic, as these scores correspond to 2 standard deviation below the mean scores of normal controls (Peretz et al., 2003; Liu et al., 2010). As shown in Table S1, 3 out of the 14 amusics (A04, A05, and A13) aged over 40 years, while the rest were between 18 and 22 years. Given that aging has been shown to affect auditory processing abilities (Chisolm et al., 2003), care was taken to find age-matched controls (C07, C08, and C10) for the three amusics in their 40s. The other controls (18–24 years) were chosen based on their MBEA global scores (all >90%) and musical training background (matched with the 14 amusics).

**Table 1** summarizes the characteristics of the amusic and control groups. As can be seen, amusics performed significantly worse than controls on all MBEA subtests. While the two groups were comparable in sex, handedness, age, and musical training background, controls received more years of education than amusics [*t*(26) = 2.31, *p* = 0.029]. This was due to the fact that the three older controls received more years of education than the three older amusics [*t*(4) = 3.29, *p* = 0.030]. When the six older participants were excluded, the difference in education background became non-significant between the two groups [amusic mean (SD) = 14.36 (2.01); control mean (SD) = 15.18 (1.72); *t*(20) = 1.02, *p* = 0.318]. In order to account for the possible contribution of education to the current results, years of education were entered as a covariate in the mixed-effects models in Section “Results.”

**Table 1:** Characteristics of the amusic (*n* = 14, 4 male and 10 female, all right-handed) and control (*n* = 14, 5 male and 9 female, all right-handed) groups.

Group	Age	Education	Musical training	Scale	Contour	Interval	Rhythm	Meter	Memory	Pitch composite	MBEA global
**Control**
Mean	25.43	16.14	1.36	27.93	27.57	27.57	29.14	27.79	29.21	83.07	94.01
SD	9.51	2.68	1.95	1.69	1.87	1.70	1.61	3.12	0.80	3.08	3.25
**Amusic**
Mean	25.50	14.07	1.07	21.93	21.57	20.00	22.93	21.14	26.71	63.50	74.60
SD	11.24	2.02	1.86	2.53	2.31	2.00	2.64	4.35	2.89	4.01	5.70
***t***-**test**
*t*	-0.02	2.31	0.40	7.39	7.55	10.80	7.51	4.65	3.12	14.48	11.07
*p*	0.986	0.029	0.694	<0.001	<0.001	<0.001	<0.001	<0.001	0.004	<0.001	<0.001

*Age, education, and musical training are in years; scores on the six MBEA subtests (scale, contour, interval, rhythm, meter, and memory) are in number of correct responses out of 30 (Peretz et al., 2003); the pitch composite score is the sum of the scale, contour, and interval scores; MBEA global score is the percentage of correct responses out of the total 180 trials; *t* is the statistic of the Welch two sample *t*-test (two-tailed, *df* = 26)*.

### STIMULI

Speech stimuli consisted of six Cantonese lexical tones spoken on the same syllable /ji/, which led to six different words in Cantonese: Tone 1, 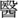, ‘doctor’; Tone 2, 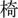, ‘chair’; Tone 3, 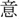, ‘meaning’; Tone 4, 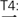, ‘son’; Tone 5, 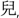, ‘ear’; Tone 6, 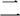 ‘two.’ Using Praat (Boersma and Weenink, 2001), a male native speaker of Hong Kong Cantonese recorded these tones onto a desktop PC in a soundproof booth, using a Shure SM10A headworn microphone and a Roland UA-55 Quad-Capture audio interface at the sampling rate of 44,100 Hz. The six original tones were then duration-normalized to 175 ms and intensity-normalized to 74 dB using Praat, so that F_0_ (fundamental frequency) was the main acoustic feature that differed across the stimuli. **Figure 1** shows time-normalized F_0_ contours of the six tones, ranging from 138–145 Hz, 107–140 Hz, 121–128 Hz, 87–99 Hz, 100–115 Hz, and 96–106 Hz, respectively.

Musical stimuli consisted of two cello tones at different pitches: high (= 150 Hz) versus low (= 112 Hz). These pitches were chosen because they approximated the highest and lowest registers of the lexical tones we used. The two cello tones were derived from a musical sound of a cello being bowed (recorded from a keyboard synthesizer; Musacchia et al., 2007), but with pitches changed to 150 and 112 Hz, duration normalized to 175 ms, and intensity normalized to 75 dB using Praat.

In the speech in noise condition, Cantonese lexical Tone 1 and Tone 6 (the same as in the speech stimuli) were presented to the participants again, but together with a recursive recording of 10-min 6-talker babble noise in Cantonese at signal-to-noise ratio (SNR) levels of 0 and 20 dB played at the background.

### PROCEDURE

All participants completed three tasks for the present study: (1) behavioral identification and (2) FFR recording of the six Cantonese tones in quiet, and (3) FFR recording of the cello tones in quiet and Cantonese Tone 1 and Tone 6 in babble noise. The three tasks normally occurred on three different days, separated by several days or months depending on the participants’ availability. The order of the three tasks was different across different participants. Participants completed the behavioral tone identification task together with other behavioral tasks (tone discrimination, tone production, and singing) for another study, which in total took about 1 h. The behavioral tone identification task was presented using E-prime 2.0 (Schneider et al., 2002), delivered through Sennheiser HD 380 PRO Headphones and a Roland UA-55 Quad-Capture audio interface at a comfortable listening level. Before the experimental trials, participants were administered a practice session consisting of two repetitions of each of the six Cantonese tones at the duration of 250 ms. In the experimental session, each of the six Cantonese tones used in the FFR task (duration = 175 ms) was presented five times in random order. Participants were required to choose the words that corresponded to the tones by clicking one of the six buttons on the computer screen as quickly and accurately as possible. Their responses and reaction times were recorded for later analysis.

Our FFR recording protocol followed closely what has been established in past research (Wong et al., 2007; Song et al., 2008; Skoe and Kraus, 2010), and various efforts had been made to exclude any potential artifacts (see Figure S5 for proof of no artifacts in a “sham” experiment). During FFR recording, participants were encouraged to rest or fall asleep in a recliner chair in an electromagnetically shielded booth with no light on. Stimuli were presented to the participants’ right ear through insert earphones (ER-3a, Etymotic Research, Elk Grove Village, IL, USA) at around 80 dB SPL, using Neuroscan Stim^2^ (Compumedics, El Paso, TX, USA). The order of the six lexical tones was counterbalanced across participants, and that of the cello tones and Tone 1 and Tone 6 in noise was fixed: Tone 1 at 20 dB SNR, Tone 1 at 0 dB SNR, Tone 6 at 20 dB SNR, Tone 6 at 0 dB SNR, cello tone at 150 Hz, and cello tone at 112 Hz. The inter-stimulus-interval jittered between 74 and 104 ms. Responses were collected using CURRY Scan 7 Neuroimaging Suite (Neuroscan, Compumedics, El Paso, TX, USA) with four Ag–AgCl scalp electrodes, differentially recorded from vertex (Cz, active) to bilateral linked mastoids (M1+M2, references), with the forehead (Fpz) as ground. Contact impedance was less than 5 kΩ for all electrodes. For each stimulus, two blocks of 1500 sweeps were collected at each polarity with a sampling rate of 20,000 Hz, lasting around 12 min.

Filtering, artifact rejection, and averaging were performed off-line using CURRY 7. Responses were band-pass filtered from 80 to 5000 Hz, 12 dB/octave, and trials with activity greater than ±35 μV were considered artifacts and rejected. Waveforms were averaged with a recording time window spanning 50 ms prior to the onset and 50 ms after the offset of the stimulus. Responses of alternating polarity were then added together to isolate the neural response by minimizing stimulus artifact and cochlear microphonic (Russo et al., 2004; Aiken and Picton, 2008).

### DATA ANALYSIS

Percentage of correct responses and reaction time were calculated for the behavioral tone identification task. Only trials with correct responses were retained in the reaction time analysis.

Main FFR data were analyzed using Matlab (The Mathworks, Natick, MA, USA) scripts adapted from the Brainstem Toolbox (Skoe et al., 2013). Before analysis, the stimuli were resampled to 20,000 Hz, to match the sampling rate of the responses. Both the stimuli and responses were band-pass filtered from 80 to 2500 Hz to remove the slower cortical ERPs and attenuate EEG noise above the limit of phase-locking (Skoe and Kraus, 2010; Bidelman et al., 2013). The FFR was assumed to encompass the entire duration of the stimulus (175 ms).

Using a sliding window analysis procedure (Wong et al., 2007; Song et al., 2008), 50-ms bins of the FFR were shifted in 1-ms steps to produce a total of 125 (= 175–50) Hanning-windowed overlapping bins in the frequency domain. A narrow-band spectrogram was calculated for each FFR bin by applying the fast Fourier transform (FFT). To increase spectral resolution, each time bin was zero-padded to 1 s before performing the FFT. The spectrogram gave an estimate of spectral energy over time and the F_0_ (pitch) contour was extracted from the spectrogram by finding the spectral peak closest to the expected (stimulus) frequency. Both F_0_ frequency and amplitude were recorded for each time bin. The same short-term spectral analysis procedure was applied to the stimulus waveforms, in order to compare the responses with the stimuli. The following time, pitch, and amplitude measurements were extracted to determine how well the amusic brainstem encodes the timing, periodicity, and the spectral envelop of the evoking stimuli compared to controls (Wong et al., 2007; Song et al., 2008; Skoe and Kraus, 2010; Skoe et al., 2013).

*Neural lag* (in ms) is the amount of time shift to achieve the maximum positive correlation between the waveforms of the stimulus and the response. This measure was calculated using a cross-correlation technique that slid the stimulus and response waveforms back and forth in time with respect to one another until the maximum positive correlation between the two was found. It estimated the FFR latency due to the neural conduction time of the auditory system for each tone/participant, which was taken into account when calculating each of the following measurements.

*Pitch strength* (or *autocorrelation*, values between –1 and 1) is a measure of periodicity and phase locking of the response. Using a short-time running autocorrelation technique, the response, in 50-ms time bins, was successively time-shifted in 1-ms steps with a delayed version of itself, and a Pearson’s *r* was calculated at each 1-ms interval. The maximum (peak) autocorrelation value was recorded for each bin, with higher values indicating more periodic time frames. *Pitch strength* was calculated by averaging the autocorrelation peaks (*r*-values) from the 125 bins for each tone/participant.

*Pitch error* (in Hz) is the average absolute Euclidian distance between the stimulus F_0_ and response F_0_ across the total of 125 time bins analyzed. It is a measure of pitch encoding accuracy of the FFR over the entire duration of the stimulus, shifted in time to match with the response based on the specific *neural lag* value obtained for each tone/participant.

*Stimulus-to-response correlation* (values between –1 and 1) is the Pearson’s correlation coefficient (*r*) between the stimulus and response F_0_ contours (shifted in time by *neural lag*). This measure indicates both the strength and direction of the linear relationship between the two signals.

*Root mean square (RMS) amplitude* (in μV) of the FFR waveform is the magnitude of neural activation over the entire FFR period (*neural lag*, *neural lag* +175 ms).

*Signal-to-noise ratio* is the ratio of the RMS amplitude of the response over the RMS of the pre-stimulus period (50 ms).

*Mean amplitudes of the first three harmonics* (in dB, indicating peak amplitudes of the power spectrum) are spectral peaks within the frequency ranges of the fundamental frequency (F_0_, first harmonic) and the next two harmonics (second and third harmonics). They were calculated by finding the largest spectral peaks in the frequency ranges of the first three harmonics in the narrow-band spectrogram after applying the short-time Fourier transform (STFT).

Statistical analyses were conducted using R (R Core Team, 2014). For parametric statistical analyses, *r*-values (pitch strength, stimulus-to-response correlation) were converted to *z′*-scores using Fisher’s transformation (Wong et al., 2007), percent correct scores for tone identification was converted using rationalized arcsine transformation (Studebaker, 1985), and reaction times were transformed using log transformation (Howell, 2009), since these measures deviated from normal distributions (Shapiro–Wilk normality test: all *p*s < 0.05). Linear mixed-effects models were fit on all measures using the R package ‘nlme’ (Pinheiro and Bates, 2009). *Post hoc* pairwise comparisons were conducted using *t*-tests with *p*-values adjusted with the Holm (1979) method.

## RESULTS

### BRAINSTEM ENCODING OF SPEECH (IN QUIET AND NOISE) AND MUSICAL STIMULI (IN QUIET)

**FIGURE 2:**
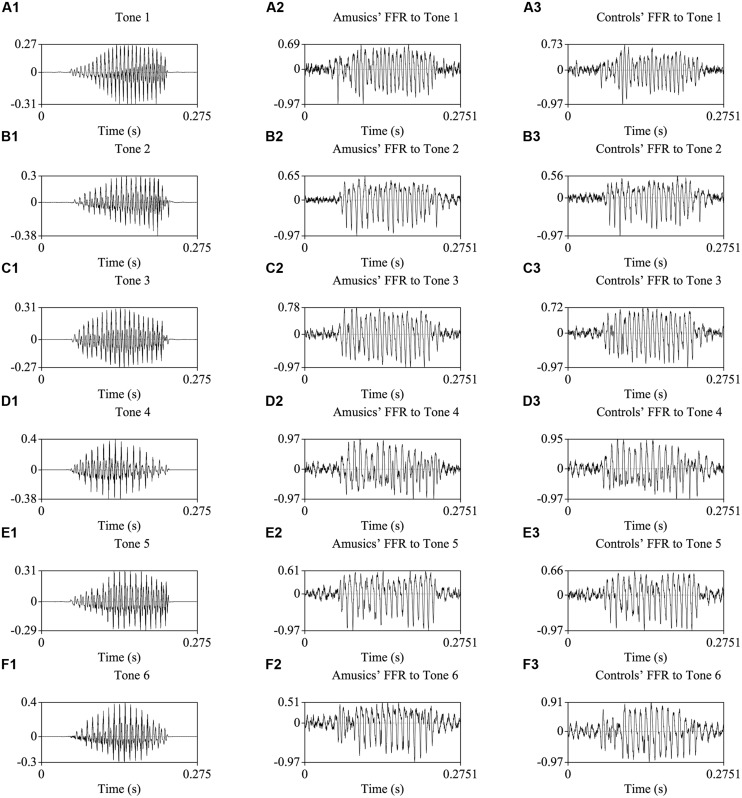
**Waveforms of the original stimuli /ji/ with six Cantonese tones (A1–F1), and those of the grand-average FFRs of amusics (A2–F2) and controls (A3–F3)**.

**FIGURE 3:**
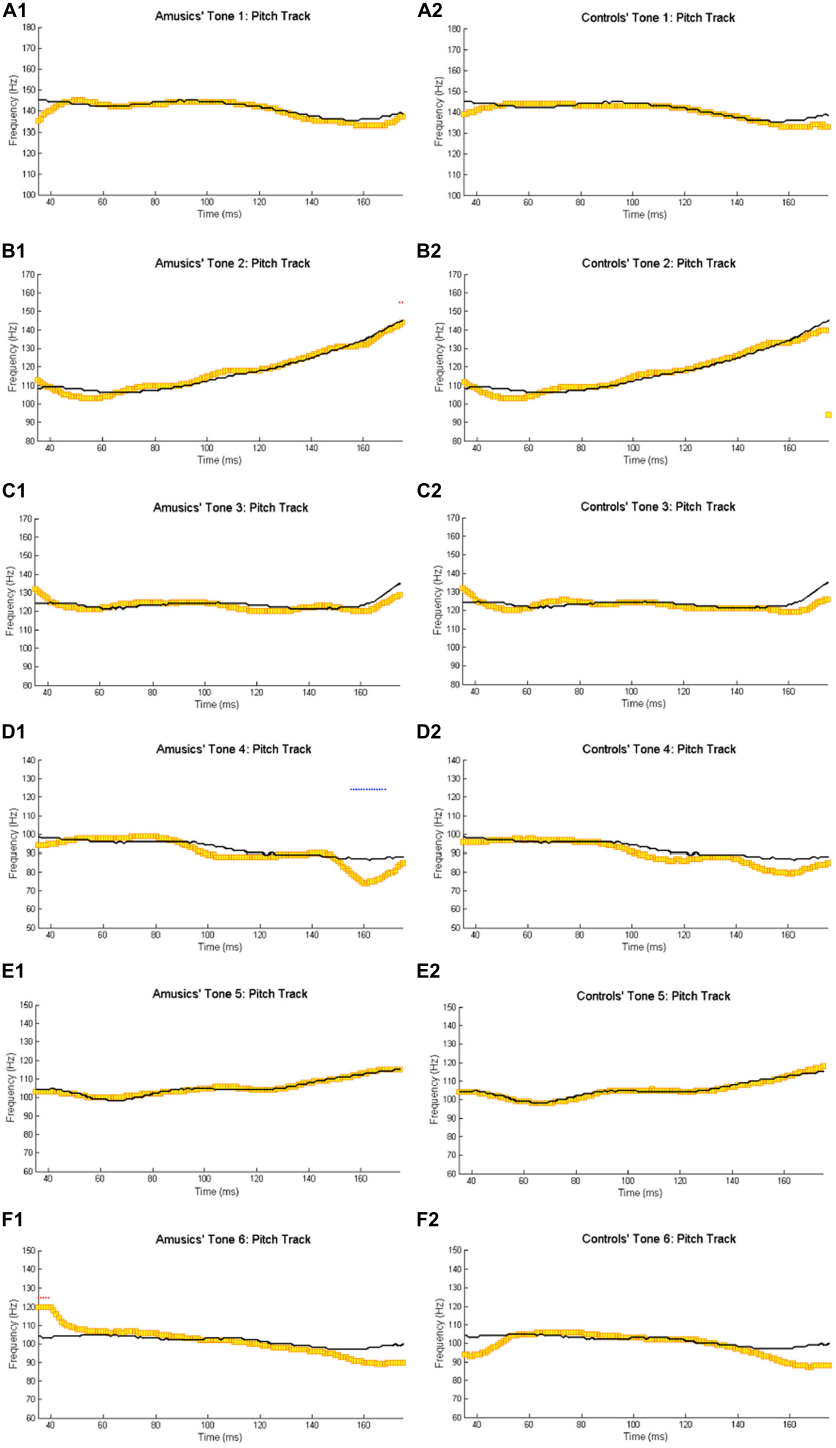
**Pitch tracks of the grand-average FFRs of amusics (A1–F1) and controls (A2–F2) to the six Cantonese tones.** The black and yellow lines indicate pitch tracks of the original stimuli and those of the responses, respectively. The small red dots signify regions where the extracted F_0_s were below the noise floor (i.e., SNR was less than one, reflecting the magnitude of the response F_0_), and the small blue dots indicate regions where the extracted F_0_s were not at the spectral maximum (indicating weak F_0_ encoding strengths; Song et al., 2008; Skoe et al., 2013).

**Figure 2** shows the waveforms of the six Cantonese lexical tones, and grand average waveforms of the FFRs to the six tones (heard in quiet) of the amusic and control groups. **Figure 3** shows pitch tracks of the original stimuli (black lines), and those of the responses (yellow lines) from both groups. In the pitch track plots, the small red dots signify regions where the extracted F_0_s were below the noise floor (i.e., SNR was less than one, reflecting the magnitude of the response F_0_), and the small blue dots indicate regions where the extracted F_0_s were not at the spectral maximum (indicating weak F_0_ encoding strengths; Song et al., 2008; Skoe et al., 2013).

**FIGURE 4:**
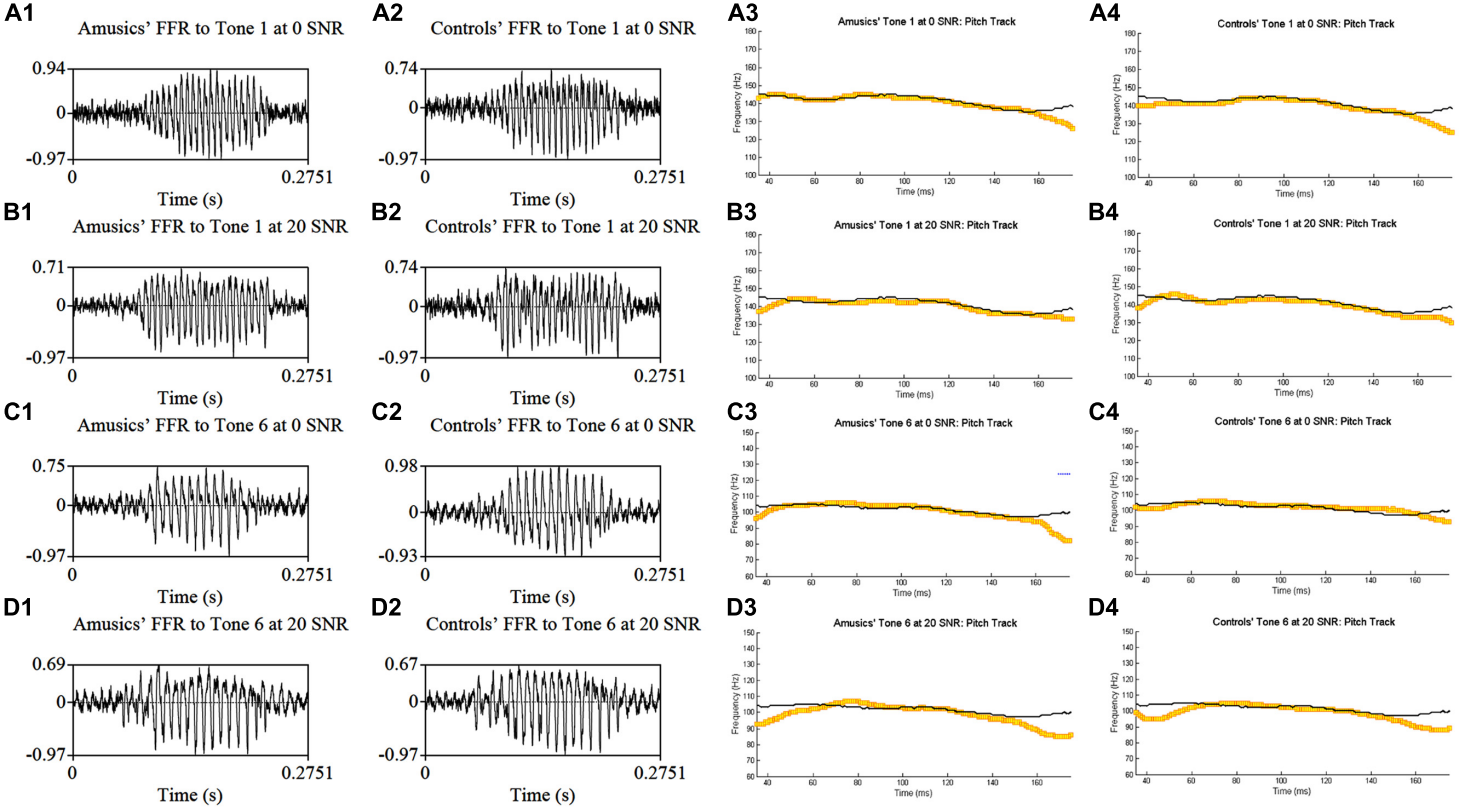
**Grand average waveforms of amusics’ (A1–D1) and controls’ (A2–D2) FFRs to Cantonese Tone 1 and Tone 6 heard in babble noise (SNR = 0 and 20 dB), and the corresponding pitch tracks (A3–D3, A4–D4)**.

**FIGURE 5:**
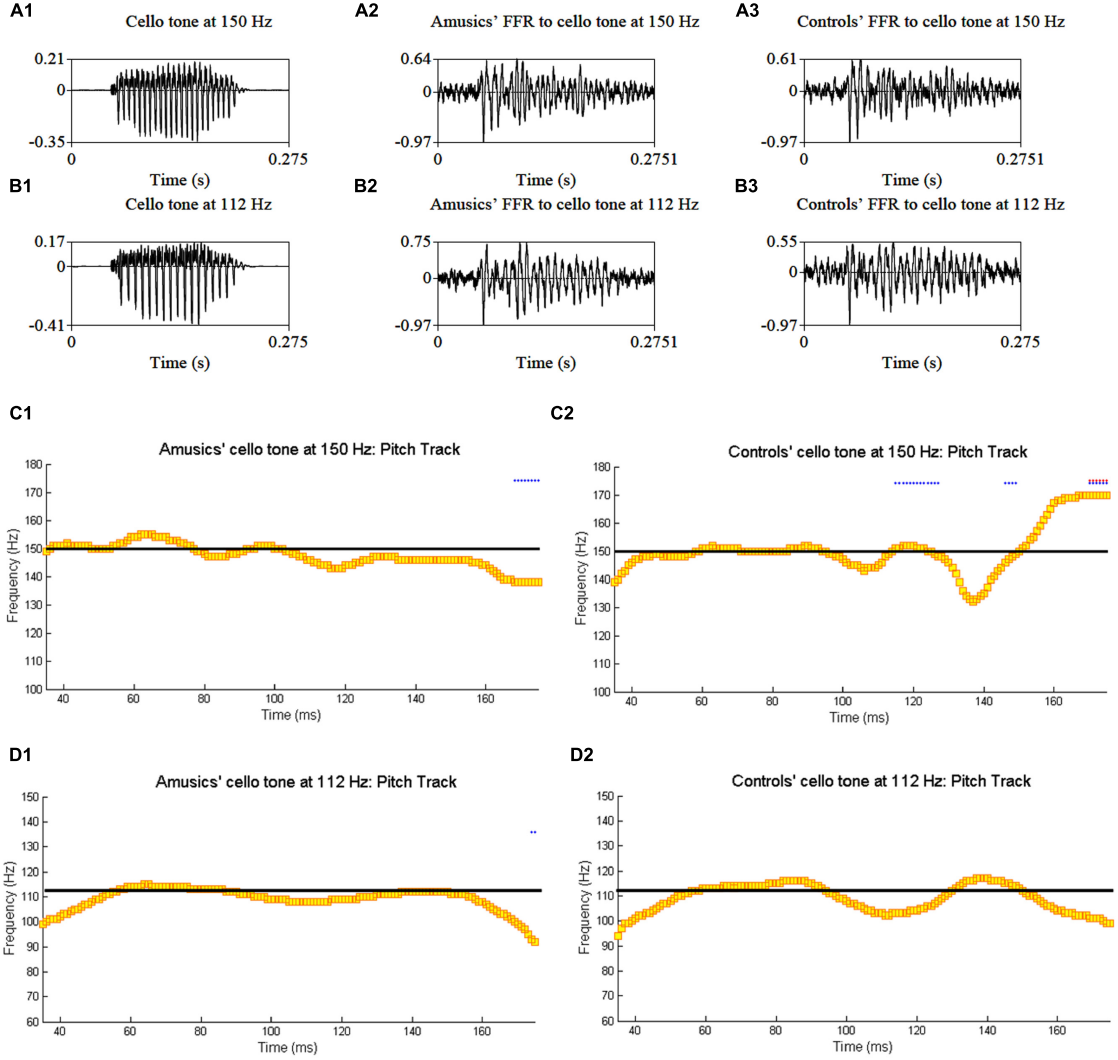
**Waveforms of the original two cello tones (A1,B1), and grand average waveforms of the FFRs to the cello tones (heard in quiet) of the amusic (A2,B2) and control (A3,B3) groups, and the corresponding pitch tracks (C1,C2,D1,D2)**.

**Figure 4** shows grand average waveforms of amusics’ and controls’ FFRs to Cantonese Tone 1 and Tone 6 heard in babble noise (SNR = 0 and 20 dB), and the corresponding pitch tracks. **Figure 5** shows the waveforms of the two cello tones, and grand average waveforms of the FFRs to the cello tones (heard in quiet) of both groups, and the corresponding pitch tracks.

Linear mixed-effects models were first fit on all FFR measures for each experimental condition (speech-in-quiet, speech-in-noise, music), with group (amusic, control), tone (Tones 1–6 in the speech-in-quiet condition, Tone 1 versus Tone 6 in the speech-in-noise condition, and high versus low cello tone in the music condition), and noise (SNR = 0 and 20 dB in the speech-in-noise condition) as fixed effects, education (in years) as a covariate, and participants (and tones nested in participants) as random effects. Detailed results are shown in Tables S2–S4.

Among all measures/conditions, a significant group effect [*F*(1,25) = 5.11, *p* = 0.033] was only observed for the amplitude of the first harmonic (F_0_) in the speech-in-noise condition, with controls showing larger F_0_ amplitudes in FFRs to tones in noise than amusics (Table S3). Although the effects of tone and noise were significant in many cases, no tone × noise or tone × noise × group interaction was observed for any measures in the speech-in-noise condition (Table S3), or tone × group interaction in the speech-in-quiet (Table S2) or music condition (Table S4). The only significant tone × group interaction [*F*(1,26) = 4.65, *p* = 0.040] was observed for neural lag in the speech-in-noise condition: controls showed longer neural lags than amusics for Tone 1 in noise, *t*(54) = –3.09, *p* = 0.003, but not for Tone 6 in noise, *t*(54) = 0.90, *p* = 0.370 (Table S3). The only significant group × noise interaction [*F*(1,52) = 7.19, *p* = 0.010] was observed for pitch error in the speech-in-noise condition, as controls showed smaller pitch errors than amusics when SNR = 0 dB, *t*(54) = 1.67, *p* = 0.101, but larger pitch errors when SNR = 20 dB, *t*(54) = –0.53, *p* = 0.597 (Table S3).

Together, these results suggest that amusics and controls showed largely comparable FFRs under different tone and noise conditions. The significant group difference in F_0_ amplitude (*p* = 0.033) and tone × group interaction on neural lag (*p* = 0.040) and group × noise interaction on pitch error (*p* = 0.010) in the speech-in-noise condition were likely due to familywise Type I errors (false positives) from multiple comparisons, as these effects would become non-significant if using Bonferroni correction to adjust for the significance level [*p* = 0.05/(9 measures × 3 conditions) = 0.0019]. Furthermore, the uneven experimental design of the tone (six levels in speech-in-quiet, two levels in speech-in-noise, and two levels in the music condition) and noise factors (two levels in speech-in-noise) gave rise to different numbers of multiple observations (replications) for each participant in each experimental condition, which would make unequal contributions to the comparison of treatments applied to different conditions (Mead, 1990). Thus, for statistical analysis and in the interest of space, FFR measures from different tones in the three experimental conditions and different noise levels in the speech-in-noise condition were pooled and averaged for each participant in the following analyses, in order to examine the overall effects of group (amusic, control) and condition (speech-in-quiet, speech-in-noise, music) on each FFR measure.

**FIGURE 6:**
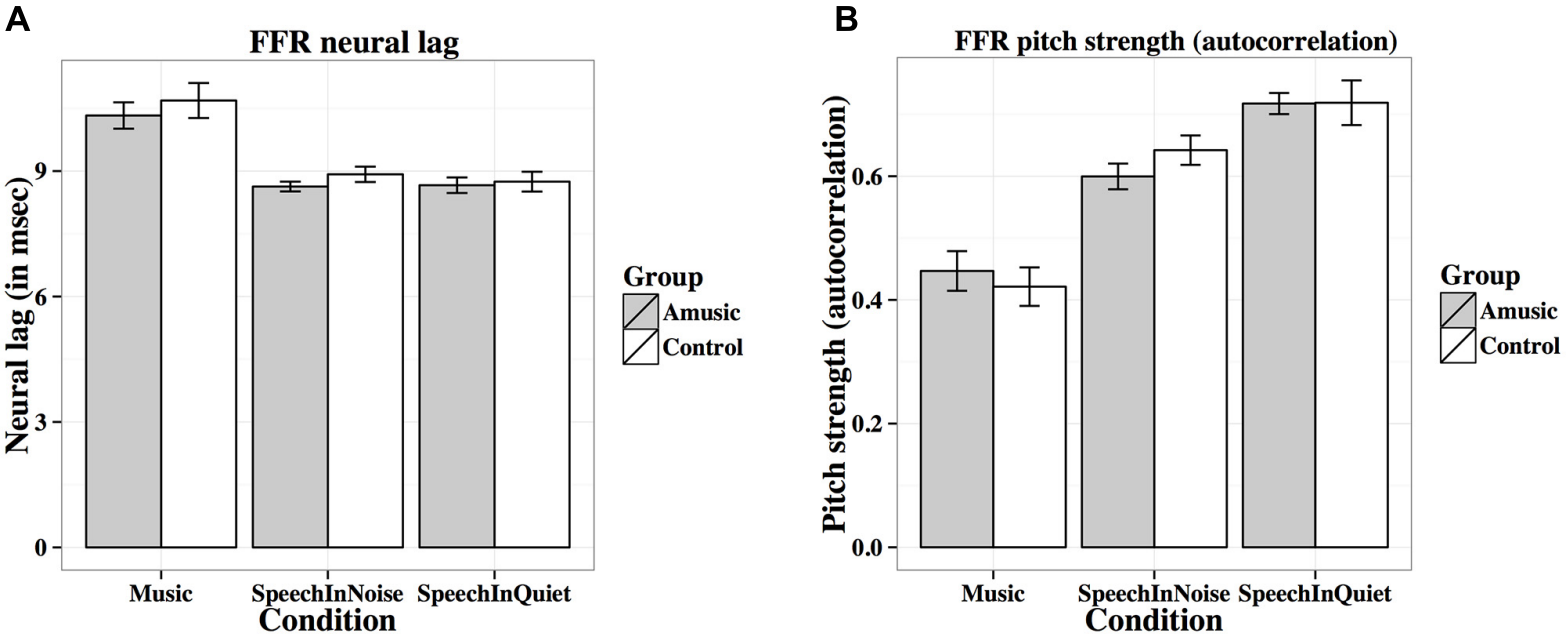
**Frequency-following response (FFR) neural lags (A; in ms) and pitch strengths (B; autocorrelations) of amusics and controls under the three experimental conditions (music, speech-in-noise, speech-in-quiet)**.

**FIGURE 7:**
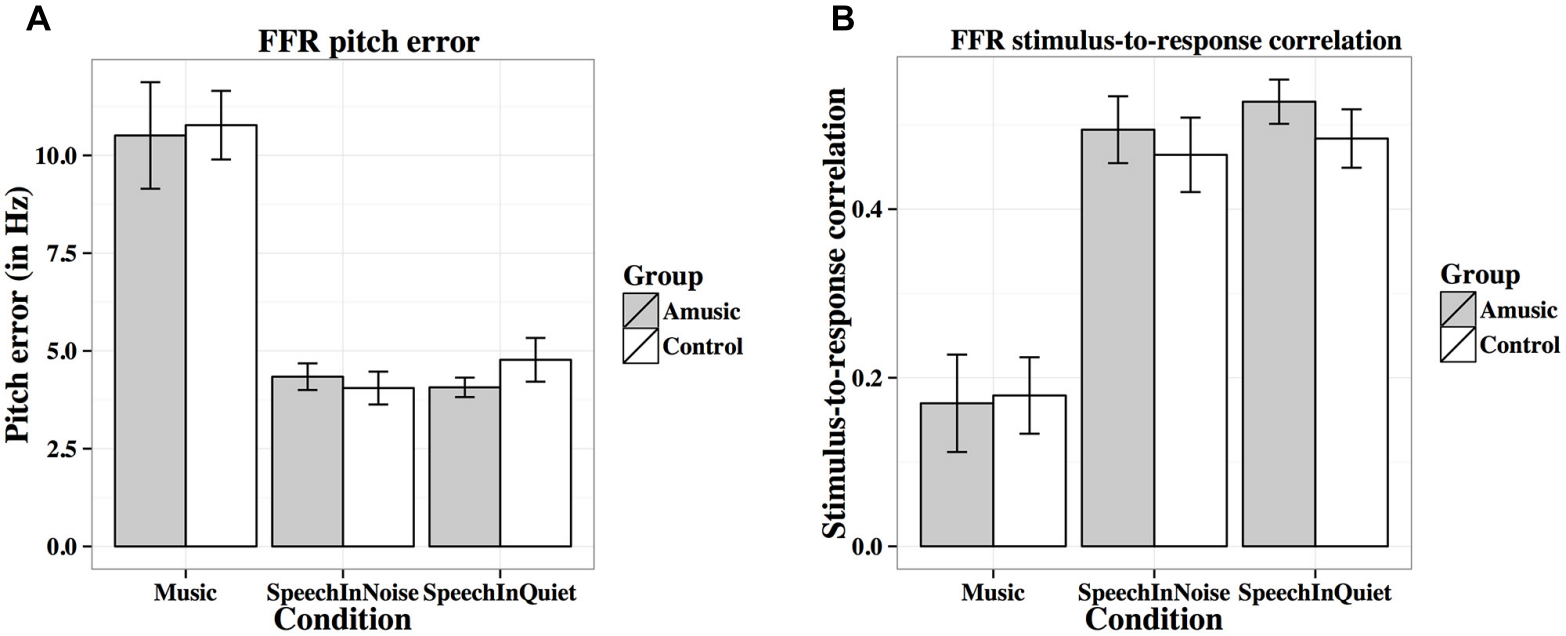
**Frequency-following response pitch errors (A; in Hz) and stimulus-to-response correlations (B) of amusics and controls under the three experimental conditions**.

**FIGURE 8:**
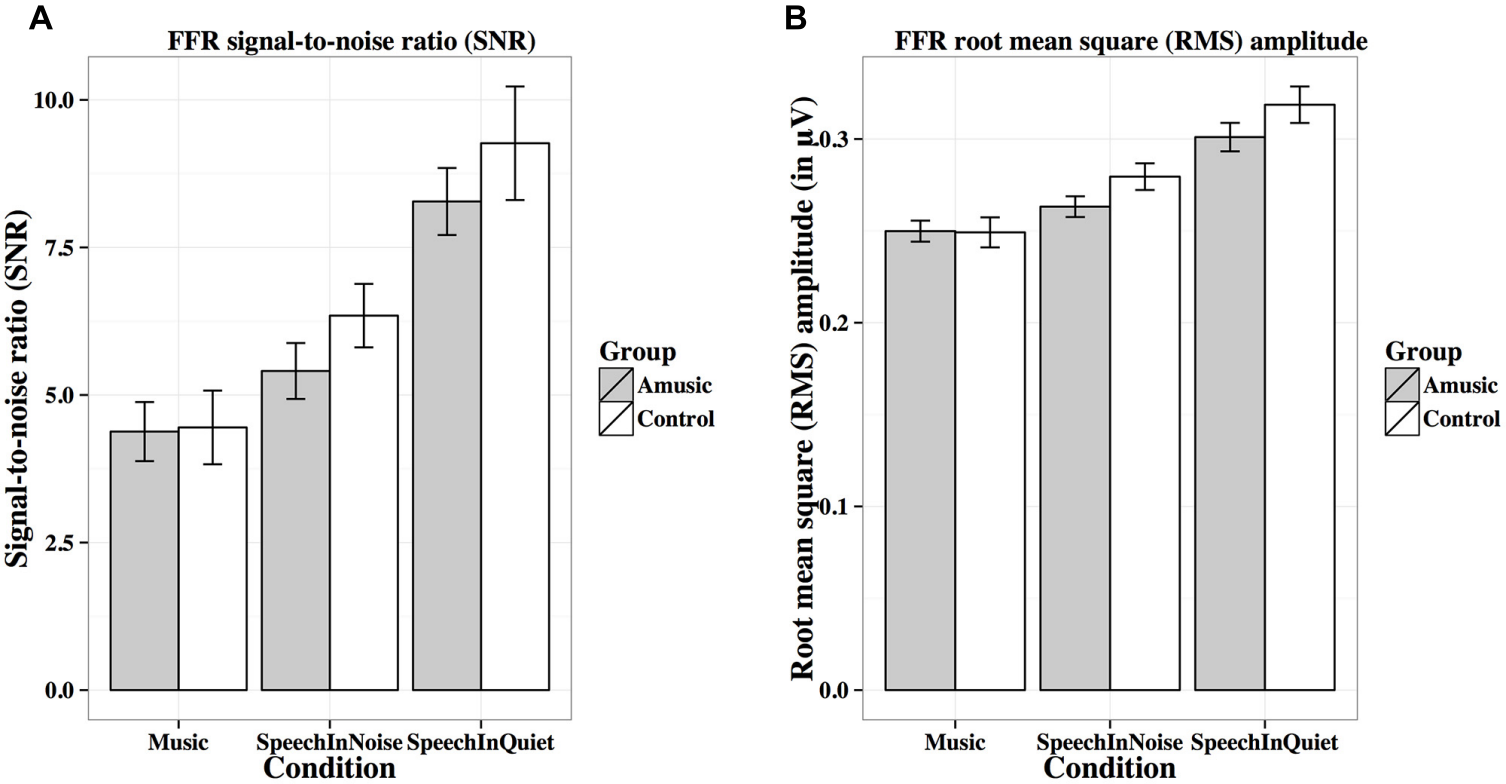
**Frequency-following response signal-to-noise ratios (A; SNRs) and root-mean-square (B; RMS) amplitudes (in μV) of amusics and controls under the three experimental conditions**.

**FIGURE 9:**
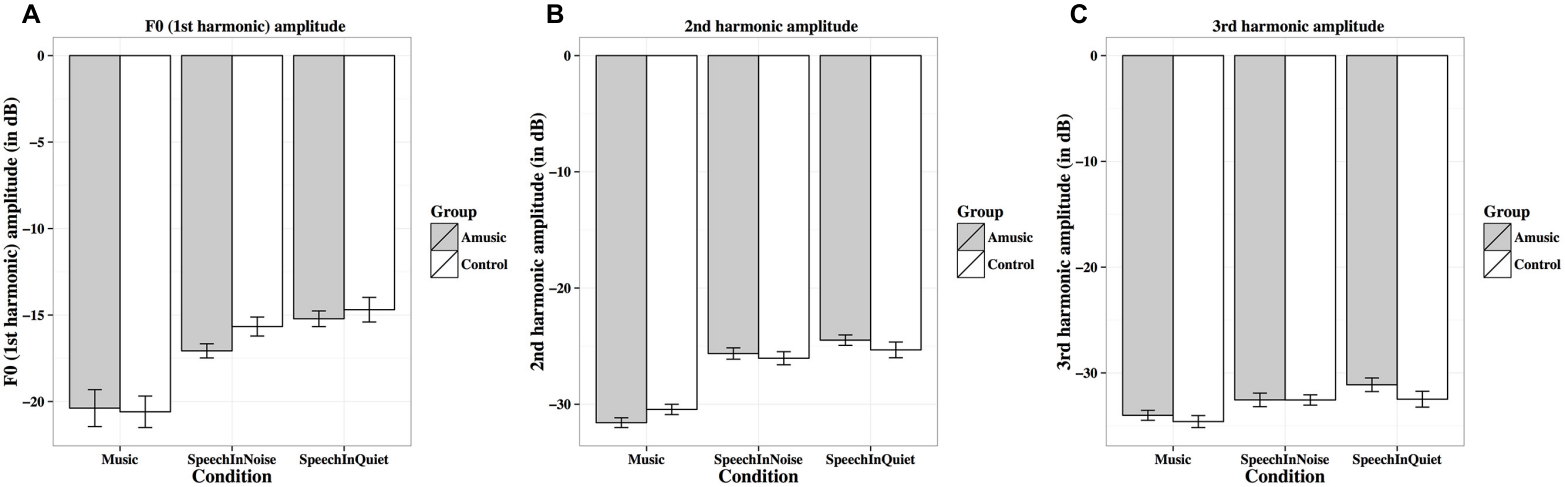
**Frequency-following response first (A), second (B), and third (C) harmonic amplitudes (in dB) of amusics and controls under the three experimental conditions**.

**Table 2** lists the results from the linear mixed-effects models on each FFR measure (averaged across different tones in each condition, and across the two noise levels in the speech-in-noise condition), with group (amusic, control) and condition (speech-in-quiet, speech-in-noise, music) as fixed effects, education (in years) as a covariate, and participants (and conditions nested in participants) as random effects. No significant effect of group or condition × group interaction was observed for any measures. The effect of condition (speech-in-quiet, speech-in-noise, music) was significant for all measures, and participants’ years of education negatively affected pitch strengths and SNRs of their FFRs. These significant effects are discussed in detail below (**Figures 6**–**9**). In the interest of space, non-significant effects are omitted from discussion.

**Table 2:** Results from the mixed-effects models on the effects of Condition [speech-in-quiet, speech-in-noise, music, *F*(2,52)], Group [amusic versus control, F(1,25)], Education [*F*(1,25)], and Condition × Group interaction [*F*(2,52)] on measures of FFRs.

Effects		Condition	Group	Education	Condition × Group
Neural lag	*F*	**32.52**	1.15	0.86	0.16
	*p*	**<0.001**	0.294	0.363	0.855
Pitch strength	*F*	**85.50**	0.40	**7.08**	0.96
	*p*	**<0.001**	0.533	**0.013**	0.388
Pitch error	*F*	**61.29**	0.10	0.54	0.28
	*p*	**< 0.001**	0.758	0.468	0.753
Stimulus-to-response correlation	*F*	**32.98**	0.47	1.69	0.07
	*p*	**<0.001**	0.500	0.205	0.932
Signal-to-noise ratio (SNR)	*F*	**56.14**	0.90	**4.96**	0.76
	*p*	**<0.001**	0.352	**0.035**	0.473
Root mean square (RMS) amplitude	*F*	**39.39**	2.44	1.34	1.10
	*p*	**<0.001**	0.131	0.257	0.340
*F*_0_ (first harmonic) amplitude	*F*	**39.34**	0.70	2.07	0.78
	*p*	**<0.001**	0.411	0.163	0.463
Second harmonic amplitude	*F*	**102.20**	0.01	0.50	2.55
	*p*	**<0.001**	0.946	0.487	0.088
Third harmonic amplitude	*F*	**21.43**	0.84	1.38	1.51
	*p*	**<0.001**	0.368	0.251	0.231

*Significant effects are highlighted in boldface*.

**Figure 6A** shows mean neural lags (in ms) of FFRs under the three experimental conditions (speech-in-quiet, speech-in-noise, music) for amusics and controls. There was a significant effect of condition (**Table 2**), as both groups exhibited longer neural lags for musical than speech-in-noise (*p* < 0.001) and speech in-quiet stimuli (*p* < 0.001).

**Figure 6B** shows mean pitch strengths (autocorrelations) of FFRs under the three experimental conditions for the two groups. The linear mixed-effects model on Fisher-transformed *z′*-scores revealed a main effect of condition (**Table 2**), as pitch strengths of both groups were the highest in the speech-in-quiet condition and the lowest in the music condition, with those of the speech-in-noise condition in between (all *p*s < 0.001). The effect of education on pitch strength was also significant (**Table 2**): the more education participants received, the lower their pitch strengths. This was confirmed by the negative correlation between participants’ education background (in years) and their pitch strengths across all conditions [*r*(82) = –0.19, *p* = 0.079].

Given that neural pitch strength of the FFR varies dramatically between steady-state and dynamic segments of the tones (Krishnan et al., 2010b; Bidelman et al., 2011a), we divided each of the tones into seven 25-ms sections and calculated mean pitch strengths of amusics and controls for each section using Krishnan et al. (2010b) and Bidelman et al. (2011a) methods. As can be seen from **Table 3**, among the total 84 (7 sections × 12 tones) pairwise comparisons, only 2 (section 1 of Tone 1 in quiet, section 3 of Tone 1 at 0 SNR) demonstrated significant group differences in pitch strength. These differences would become non-significant if using Bonferroni correction to adjust for the significance level (*p* = 0.05/84 = 0.0006) in order to prevent familywise Type I errors (false positives). In general, these results suggest that amusics did not differ from controls in FFR pitch strength across different sections of the tones in speech-in-quiet, speech-in-noise, or music conditions.

**Table 3:** Mean pitch strengths of amusics and controls over seven 25-ms sections of the six Cantonese tones heard in quiet, Cantonese Tone 1 and Tone 6 heard in noise (SNR = 0 and 20 dB), and cello tones at 150 and 112 Hz.

Tone	Group	Section (each 25 ms)						
		1	2	3	4	5	6	7
T1 in quiet	Amusic	**0.09**	0.44	0.45	0.54	0.51	0.57	0.43
	Control	**0.27**	0.46	0.37	0.50	0.56	0.54	0.43
	*p*	**0.0047**	0.75	0.31	0.56	0.40	0.55	0.94
T2 in quiet	Amusic	0.39	0.52	0.51	0.57	0.57	0.59	0.17
	Control	0.32	0.52	0.46	0.54	0.56	0.57	0.21
	*p*	0.24	0.98	0.21	0.40	0.69	0.62	0.59
T3 in quiet	Amusic	0.34	0.52	0.55	0.58	0.58	0.51	0.43
	Control	0.28	0.51	0.58	0.56	0.56	0.52	0.48
	*p*	0.39	0.89	0.49	0.80	0.66	0.84	0.31
T4 in quiet	Amusic	0.44	0.51	0.48	0.49	0.47	0.43	0.35
	Control	0.40	0.46	0.48	0.49	0.49	0.40	0.33
	*p*	0.45	0.30	0.88	0.93	0.67	0.48	0.51
T5 in quiet	Amusic	0.41	0.45	0.48	0.49	0.49	0.55	0.51
	Control	0.38	0.52	0.50	0.55	0.51	0.54	0.57
	*p*	0.50	0.17	0.73	0.08	0.67	0.70	0.18
T6 in quiet	Amusic	0.15	0.38	0.52	0.57	0.52	0.52	0.39
	Control	0.19	0.41	0.54	0.54	0.51	0.49	0.35
	*p*	0.54	0.55	0.70	0.45	0.64	0.50	0.37
T1 at 0 SNR	Amusic	0.13	0.32	**0.42**	0.51	0.52	0.45	0.28
	Control	0.14	0.37	**0.53**	0.57	0.56	0.52	0.33
	*p*	0.76	0.37	**0.0499**	0.34	0.50	0.21	0.39
T1 at 20 SNR	Amusic	0.24	0.46	0.46	0.51	0.51	0.51	0.46
	Control	0.16	0.44	0.45	0.51	0.53	0.58	0.47
	*p*	0.21	0.67	0.83	0.93	0.77	0.17	0.74
T6 at 0 SNR	Amusic	0.18	0.39	0.49	0.52	0.49	0.42	0.22
	Control	0.19	0.38	0.52	0.53	0.51	0.46	0.30
	*p*	0.85	0.89	0.46	0.69	0.28	0.31	0.07
T6 at 20 SNR	Amusic	0.13	0.37	0.51	0.56	0.50	0.47	0.39
	Control	0.13	0.32	0.54	0.52	0.50	0.47	0.33
	*p*	0.97	0.30	0.26	0.22	0.93	0.88	0.08
Cello tone at 150 Hz	Amusic	0.14	0.38	0.18	0.31	0.26	0.31	0.10
	Control	0.13	0.36	0.30	0.27	0.17	0.28	0.19
	*p*	0.81	0.78	0.14	0.65	0.20	0.67	0.16
Cello tone at 112 Hz	Amusic	0.13	0.38	0.36	0.44	0.37	0.38	0.14
	Control	0.18	0.30	0.32	0.35	0.31	0.33	0.15
	*p*	0.41	0.24	0.53	0.16	0.28	0.51	0.79

*The *p*-values were obtained from Welch two sample *t*-tests (two-tailed, *df* = 26). Sections with significant group differences are highlighted in boldface. Note that the pitch strength values in this table are different from those in **Figure 6B** due to different calculation methods used (Krishnan et al., 2010b; Bidelman et al., 2011a; Skoe et al., 2013)*.

**Figure 7A** shows mean pitch errors (in Hz) of FFRs under the three experimental conditions for amusics and controls. There was a significant effect of condition (**Table 2**), as both groups showed larger pitch errors for musical than speech-in-noise and speech-in-quiet stimuli (both *p*s < 0.001).

**Figure 7B** shows mean stimulus-to-response correlations of the three types of stimuli for the two groups. The linear mixed-effects model on Fisher-transformed *z′*-scores revealed a main effect of condition (**Table 2**), as both groups demonstrated lower stimulus-to-response correlations for musical than speech-in-noise and speech-in-quiet stimuli (both *p*s < 0.001), and lower stimulus-to-response correlations for speech-in-noise than speech-in-quiet stimuli (*p* = 0.048).

**Figure 8A** shows mean SNRs of FFRs under the three experimental conditions for amusics and controls. SNR differed significantly across different experimental conditions (**Table 2**), in the order of music < speech-in-noise < speech-in-quiet for both groups (all *p*s < 0.05). There was also a significant effect of education on SNR (**Table 2**): the more education participants received, the lower their SNRs. This was confirmed by the negative correlation between participants’ education background (in years) and their SNRs across all conditions [*r*(82) = –0.20, *p* = 0.075].

**Figure 8B** shows mean RMS amplitudes (in μV) of the FFR waveforms under the three experimental conditions for amusics and controls. RMS amplitudes differed significantly across different experimental conditions (**Table 2**), in the order of music < speech-in-noise < speech-in-quiet for both groups (all *p*s < 0.01).

**Figure 9** shows mean amplitudes of spectral peaks within the frequency ranges of the fundamental frequency (F_0_, first harmonic) and the next two harmonics (second and third harmonics) under the three experimental conditions for amusics and controls. Mean amplitudes of these harmonics were significantly affected by different experimental conditions (**Table 2**), as both groups showed lower harmonic amplitudes for music than speech-in-noise/speech-in-quiet stimuli (all *p*s < 0.01).

### BEHAVIORAL IDENTIFICATION OF THE SIX CANTONESE LEXICAL TONES

**FIGURE 10:**
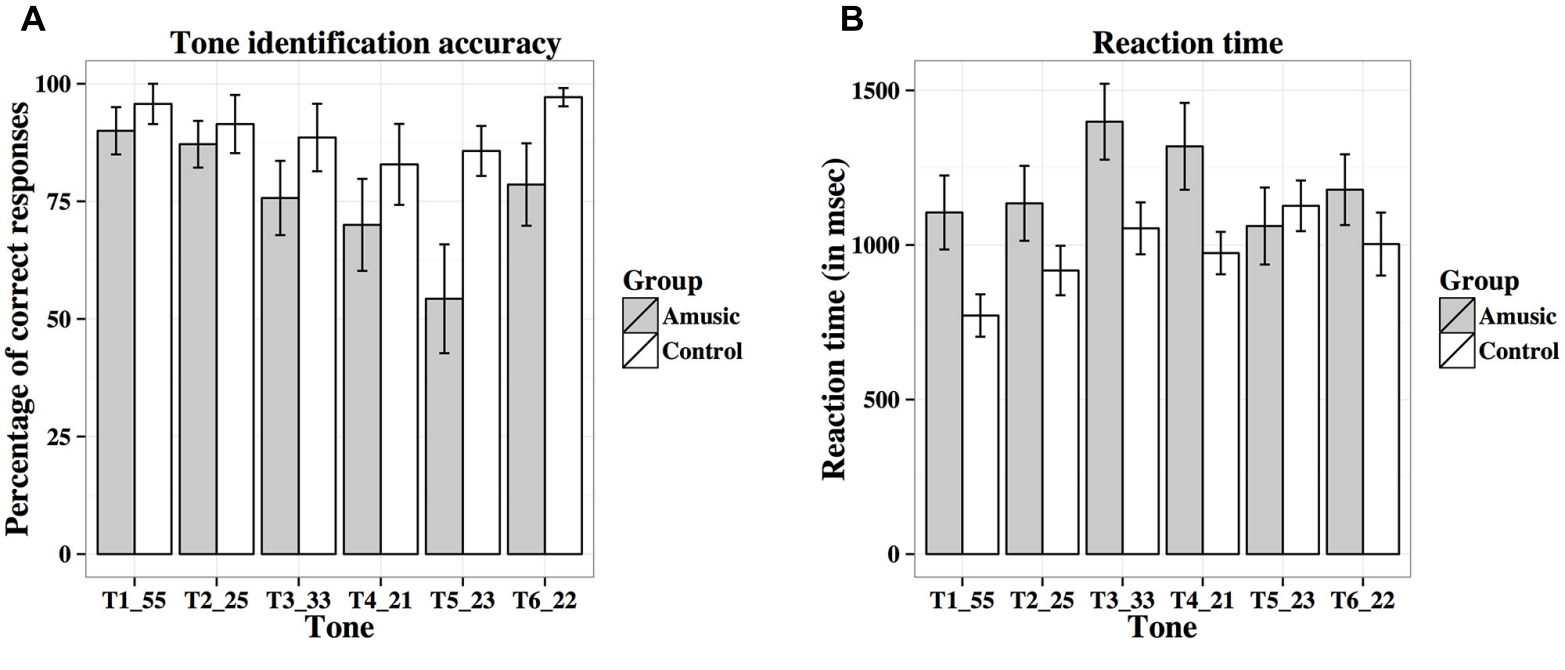
**Percent-correct scores (A) and reaction times (B; in ms) of amusics and controls for the behavioral tone identification task**.

**Figure 10A** shows percent-correct scores of amusics and controls for the behavioral tone identification task. A linear mixed-effects model was fit on rationalized arcsine transformed scores, with group (amusic, control) and tone (Tones 1–6) as fixed effects, education (in years) as a covariate, and participants (and tones nested in participants) as random effects. Tone identification scores differed significantly across different tones [*F*(5,130) = 4.64, *p* = 0.002 with the Greenhouse–Geisser correction], with Tone 5 receiving significantly worse identification than Tone 1 (*p* = 0.025). There was a significant main effect of group [*F*(1,25) = 4.81, *p* = 0.038], as controls achieved better tone identification performance than amusics. A significant effect of education was also observed for tone identification [*F*(1,25) = 4.73, *p* = 0.039]: the more education participants received, the worse their tone identification. This was confirmed by the negative correlation between participants’ education background (in years) and their mean tone identification scores across the six tones [*r*(26) = –0.18, *p* = 0.351]. No significant tone × group interaction was observed [*F*(5,130) = 1.15, *p* = 0.337 with the Greenhouse–Geisser correction].

**Figure 10B** shows reaction times of amusics and controls on the correct trials for the identification of the six tones. Among the 28 participants, five participants (four amusics and one control) scored 0% correct on some tones (Tones 3–5), thus leading to missing data. The linear mixed-effects model on log-transformed reaction times indicated that reaction times differed significantly across different tones [*F*(5,123) = 3.77, *p* = 0.003; or *F*(5,105) = 3.01, *p* = 0.016 with the Greenhouse–Geisser correction and when participants with missing data were removed], as Tone 1 elicited significantly shorter reaction times than Tones 3–5 (all *p*s < 0.05). The other effects were non-significant [Group: *F*(1,25) = 1.52, *p* = 0.229; Education: *F*(1,25) = 0.03, *p* = 0.856; Tone × Group: *F*(5,123) = 1.00, *p* = 0.423; or *F*(5,105) = 0.83, *p* = 0.522 with the Greenhouse–Geisser correction and when participants with missing data were removed].

**Table 4** shows confusion matrices of tone identification of the two groups. Fisher’s Exact Test for Count Data (two-tailed) revealed significant differences in confusion patterns between amusics and controls for Tone 4 (*p* = 0.006), Tone 5 (*p* < 0.001), and Tone 6 (*p* = 0.012). Amusics were more likely than controls to confuse between tones that shared similar acoustic features, e.g., Tones 2 and 5 (two rising tones), Tones 3 and 6 (two level tones), and Tones 4 and 6 (two low tones).

**Table 4:** Confusion matrices of tone identification of amusics and controls.

	T1_55	T2_25	T3_33	T4_21	T5_23	T6_22
**Amusic**
T1_55	**90.00**	0.00	7.14	0.00	0.00	0.00
T2_25	1.43	**87.14**	4.29	2.86	40.00	4.29
T3_33	4.29	0.00	**75.71**	7.14	2.86	7.14
T4_21	0.00	1.43	0.00	**70.00**	1.43	7.14
T5_23	1.43	10.00	4.29	2.86	**54.29**	0.00
T6_22	0.00	1.43	7.14	17.14	1.43	**78.57**
No response	2.86	0.00	1.43	0.00	0.00	2.86
**Control**
T1_55	**95.71**	0.00	7.14	7.14	0.00	0.00
T2_25	0.00	**91.43**	1.43	0.00	12.86	0.00
T3_33	4.29	0.00	**88.57**	1.43	0.00	1.43
T4_21	0.00	0.00	0.00	**82.86**	0.00	1.43
T5_23	0.00	8.57	0.00	0.00	**85.71**	0.00
T6_22	0.00	0.00	1.43	8.57	1.43	**97.14**
No response	0.00	0.00	1.43	0.00	0.00	0.00

### CORRELATION ANALYSES

Correlation analyses were conducted between brainstem encoding and behavioral identification of the six Cantonese lexical tones heard in quiet in order to examine whether there was any association between neural and cognitive processing of lexical tones in the current participants. Results revealed no significant correlation between FFR pitch measures (pitch strength, pitch error, stimulus-to-response correlation) and tone identification accuracy, or between FFR neural lag and tone identification response time when all participants were analyzed together or for each group alone (all *p*s > 0.05).

Correlation analyses were conducted between MBEA global scores and FFR/behavioral measures of lexical tone processing in quiet in order to examine whether there was any association between music perception and lexical tone processing in the current participants. A significant positive correlation was observed between MBEA global scores and mean rationalized arcsine transformed tone identification scores when both groups were combined [*r*(26) = 0.56, *p* = 0.002] and for the control group [amusics: *r*(12) = 0.53, *p* = 0.051; controls: *r*(12) = 0.71, *p* = 0.005]: the better the music perception, the better the lexical tone identification. There was also a significant negative correlation between MBEA global scores and mean amplitudes of the third harmonic of FFRs to the speech-in-quiet stimuli (averaged across the six tones) for the control group [*r*(12) = –0.63, *p* = 0.016): the better the music perception, the smaller the amplitude of the third harmonic of FFRs to the speech-in-quiet stimuli.

Correlation analyses between MBEA global scores and FFR measures of speech-in-noise stimuli revealed significant correlations in several cases for the control group only. First, significant correlations were observed between MBEA global scores and pitch strengths [*r*(12) = 0.77, *p* = 0.001], pitch errors [*r*(12) = –0.60, *p* = 0.021], SNRs [*r*(12) = 0.66, *p* = 0.010], and RMS amplitudes [*r*(12) = 0.58, *p* = 0.030] of FFRs to speech-in-noise stimuli in controls. Second, there was also a significant positive correlation between MBEA global scores and mean amplitudes of the first harmonic of the speech-in-noise stimuli for the control group [*r*(12) = 0.61, *p* = 0.020].

Correlation analyses between MBEA global scores and FFR measures of cello tones revealed significant correlations in several cases for the control group only. First, positive correlations were observed between MBEA global scores and pitch strengths [*r*(12) = 0.57, *p* = 0.032] and SNRs [*r*(12) = 0.63, *p* = 0.015] of FFRs to cello tones in controls. Second, there was also a significant negative correlation between MBEA global scores and mean amplitudes of the third harmonic of the cello stimuli for the control group [*r*(12) = –0.81, *p* < 0.001].

### POWER ANALYSIS

Power analysis was conducted in order to examine whether the current non-significant group differences in brainstem encoding of speech (in quiet and noise) and musical stimuli were due to sample size or the power of this study. According to Cohen (1988), a study should have at least 80% power to be worth doing. Suppose we wanted to achieve a medium effect size (Cohen’s *d* = 0.5) with significance level at *p* < 0.05, having *n* = 28 participants would give us a power of 84% to find amusics being worse than controls in the tasks. Indeed, the significant group difference in behavioral identification of the six Cantonese tones showed a medium effect size (Cohen’s *d* = 0.508, on rationalized arcsine transformed scores) in the current study. Therefore, the current finding of intact brainstem encoding of speech (in quiet and noise) and musical stimuli in amusia is unlikely to be inconclusive.

## DISCUSSION

This study investigated the relationship between brainstem representation and behavioral identification of lexical tones as well as brainstem encoding of speech-in-noise and musical stimuli in Cantonese-speaking individuals with congenital amusia, a disorder of pitch processing in music and speech. Measurements of the FFR waveforms revealed no evidence of abnormal brainstem encoding of speech (in quiet and noise) or musical stimuli for amusics relative to controls, in terms of timing, frequency, and amplitude. However, amusics performed significantly worse than controls on identification of lexical tones. The dissociation between brainstem representation and behavioral identification of lexical tones was further confirmed by the lack of correlation between FFR and behavioral pitch and timing measures. No correlation was observed between amusics’ music perception scores and FFR measures of speech-in-noise/musical stimuli, either. These findings suggest that amusics’ subcortical neural responses simply represent acoustic/sensory properties of the speech or musical stimuli, rather than reflecting their higher-level pitch-processing deficits.

Brainstem responses to speech/music sounds have been considered as biological markers of individuals’ auditory, music, and language processing abilities (Chandrasekaran and Kraus, 2010; Skoe and Kraus, 2010). Previous neuroimaging and neurophysiological studies of amusia suggest that amusics’ pitch processing deficits may or may not start in the auditory cortex (Albouy et al., 2013a; Peretz, 2013). Given the positive association between the quality of brainstem representation of speech/music sounds and musical expertise (Musacchia et al., 2007; Wong et al., 2007; Parbery-Clark et al., 2009a; Strait et al., 2012), it would be worth exploring whether disordered musical functioning in amusia is related to impaired subcortical representation of pitch-bearing information. Our results revealed no evidence of abnormal brainstem representation of speech (in quiet and noise) or musical stimuli in amusia, across all FFR measures in terms of timing, frequency, and amplitude, suggesting that amusics’ pitch processing deficits are unlikely to originate from the auditory brainstem. It has been proposed that the top–down corticofugal pathway may be a potential mechanism for explaining brainstem encoding advantage in tone language speakers and musicians (Chandrasekaran and Kraus, 2010). Our current results indicate that if in fact the previous studies can be explained by the corticofugal system, it is not in play in amusics, which suggests that amusics may have a very high level of deficits that are confined within the cortex.

Compared to non-musicians, musicians have been shown to have enhanced brainstem encoding of speech and musical stimuli in a quiet setting (Musacchia et al., 2007). If subcortical encoding of speech/music sounds reflected musical aptitude along the entire spectrum from musicians to non-musician controls and to amusics, we would expect amusics to show impaired brainstem encoding of speech and musical stimuli compared to controls. However, our results indicate normal brainstem encoding of speech and musical stimuli in quiet in Cantonese-speaking amusics, across six different lexical tones and two cello tones (**Figures 2**–**3** and **5**; Tables S2 and S4). This suggests that amusics’ speech and musical processing deficits are not due to reduced brainstem encoding of speech and musical sounds.

Previous research also suggests that musicians have enhanced brainstem encoding of speech in noise, which is coupled with perceptual enhancement in hearing speech in noise (Parbery-Clark et al., 2009a; Strait et al., 2012). The strong association between brainstem representation of F_0_ in noise and perceptual performance on speech-in-noise has also been observed in English-speaking non-musicians (Song et al., 2011). While it is a matter of debate whether musicians indeed have enhanced ability to understand speech in noise compared to non-musicians (Parbery-Clark et al., 2009b; Ruggles et al., 2014), Mandarin-speaking amusics have shown reduced speech intelligibility in both quiet and noise, with natural or flattened F_0_, relative to normal controls (Liu et al., 2015). Although our current study did not measure participants’ behavioral performance on understanding speech in noise, our brainstem data (**Figure 4**; Table S3) revealed largely normal FFRs to speech in noise in Cantonese-speaking amusics as compared to controls, but with two exceptions. First, controls showed larger first harmonic (F_0_) amplitudes in FFRs to speech in noise than amusics [*F*(1,25) = 5.11, *p* = 0.033; Table S3], indicating stronger subcortical spectral encoding of speech in noise in controls relative to amusics. However, amusics demonstrated shorter neural lags than controls in FFRs to Tone 1 in noise [*t*(54) = –3.09, *p* = 0.003; Table S3], suggesting shorter neural conduction time for speech in noise in amusics versus controls. These mixed results, although interesting, may be due to familywise Type I errors (false positives) from multiple comparisons, as no significant group difference was observed in the overall ANOVAs using measures averaged across different tone and noise conditions (**Table 2**). Further studies are required to examine the relationship between amusics’ speech comprehension deficits in quiet and noise and their subcortical and cortical representation of speech in quiet and noise.

Despite demonstrating largely normal brainstem encoding of speech and musical stimuli, a deficit in lexical tone identification was observed for the current sample of amusics. This is consistent with previous findings of impaired lexical tone processing in Mandarin-speaking amusics (Nan et al., 2010; Jiang et al., 2012b; Liu et al., 2012a). For Cantonese, it has been suggested that tones with similar acoustic features, e.g., Tones 2 and 5 (two rising tones), Tones 3 and 6 (two level tones), and Tones 4 and 6 (two low tones), are in the process of merging into one single category due to the ongoing sound change (Mok and Zuo, 2012; Law et al., 2013; Mok et al., 2013). In our current tone identification task, confusion matrices of amusics and controls suggested that amusics were more likely than controls to confuse between these acoustically similar tones, presumably due to their pitch discrimination difficulty.

In line with previous findings of different performance of amusics on implicit/pre-attentive versus explicit/attentive tasks and conditions (Loui et al., 2008; Peretz et al., 2009; Liu et al., 2010; Hutchins and Peretz, 2012; Mignault Goulet et al., 2012; Omigie et al., 2012; Moreau et al., 2013), the current study observed a dissociation between pre-attentive subcortical representation and perceptual identification of lexical tones in amusia. This suggests that amusics have difficulty mapping tonal patterns onto long-time stored linguistic categories, despite having received sufficient acoustic input of these tones at the brainstem level. This dissociation suggests that amusia is a higher-level pitch-processing disorder. It has been shown that higher-level processing of internal representations of linguistic tones is implicated in the left inferior frontal gyrus (Hsieh et al., 2001). Thus, amusics’ tone identification deficits may be related to their structural abnormality in this brain region (Mandell et al., 2007). Further studies are required to examine how amusic tone-language speakers map tonal patterns onto internalized linguistic representations during speech comprehension.

Previous research has led to mixed results regarding the relationship between neurophysiological and behavioral processing of complex sounds. In Bidelman et al. (2011b), Chinese listeners demonstrated musician-like, enhanced brainstem encoding of musical pitch compared to non-musicians, but similar to non-musicians, they did not achieve musician-level performance on musical pitch discrimination. Similarly, compared to English musicians and non-musicians, Chinese listeners showed larger MMN (mismatch negativity) responses to the within-category difference between their native curvi-linear rising tone and a strictly linear rising pitch, although they were not as accurate as those non-native listeners in discriminating these within-category tones (Chandrasekaran et al., 2009). Nevertheless, several other studies have reported significant correlations between brainstem responses and behavioral measures such as frequency discrimination (Bidelman and Krishnan, 2010; Krishnan et al., 2010a, 2012; Carcagno and Plack, 2011; Marmel et al., 2013) and hearing speech in noise (Parbery-Clark et al., 2009a; Song et al., 2011; Strait et al., 2012). It is possible that the dissociation between neural and behavioral pitch processing in Chinese listeners observed in previous studies was due to the nature of the stimuli used: unfamiliar musical pitches (Bidelman et al., 2011b) and within-category tones (Chandrasekaran et al., 2009), which would naturally lead to inferior performance of Chinese listeners (non-musicians) as compared to musicians and non-native listeners. Using natural and behaviorally relevant lexical tones as stimuli, which were neither unfamiliar nor within-category for native Cantonese listeners, the current study excluded the confounding factor of stimuli but reached similar findings as in previous studies (Chandrasekaran et al., 2009; Bidelman et al., 2011b). Our finding of the brain-behavior dissociation in the processing of lexical tones in amusia requires further investigations into the interplay between subcortical and cortical structures along the auditory pathway, as our current results seem to suggest that, although the amusic brainstem maintains detailed representations of lexical tones, it does not lead to perceptual recognition of these tones at the normal level.

A growing number of reports have suggested that FFR is not a direct correlate of the pitch percept itself, but only reflects exogenous stimulus properties, e.g., temporal information originated from the auditory periphery (Bidelman et al., 2011b, 2013; Gockel et al., 2011; Plack et al., 2014). On the other hand, the human “pitch center,” which is located in lateral Heschl’s gyrus (HG) and anterolateral PT (planum temporale) of the auditory cortex, has been proposed to represent the percept of pitch (Bendor, 2012; Griffiths and Hall, 2012; Plack et al., 2014). Our findings seem to agree with these proposals, as amusics’ pitch-processing deficits were not reflected in the brainstem, but were present at the perceptual/behavioral level in acoustic-to-phonetic mapping of lexical tones. A recent study examining categorical perception of vowels also showed that cortical but not brainstem speech representations accounted for the acoustic-to-phonetic mapping of speech sounds (Bidelman et al., 2013).

Finally, it is worth mentioning that the current amusics, being exposed to six lexical tones in Cantonese, which is rare in the world’s tone languages (Yip, 2002), may have developed a protective mechanism against brainstem deficits through development, especially in terms of pitch encoding. Although subtle spectral encoding deficits (F_0_ amplitude) for speech in noise were observed for the current Cantonese-speaking amusics (but note amusics’ quicker neural conduction time than controls for Tone 1 in noise; Table S3), it is possible that if we examined amusics from non-tonal language backgrounds (e.g., English, French), we would see salient pitch-encoding deficits in the amusic brainstem. However, a recent poster investigating brainstem responses to dissonance in musical stimuli in French-speaking amusics (Cousineau et al., 2014) also observed a trend for normal brainstem encoding of musical dissonance in amusia. Furthermore, a recent psychoacoustic study reported that amusics showed normal spectral and temporal coding of pitch in the auditory periphery (Cousineau et al., 2015). Further studies are required to confirm these observations, and explore how the amusic brainstem encodes more complex musical stimuli compared to normal controls, using participants from both tone and non-tonal language backgrounds.

In summary, the current study revealed a dissociation between subcortical representation (normal) and behavioral identification (impaired) of lexical tones as well as intact brainstem encoding of speech (in quiet and noise) and musical stimuli in Cantonese-speaking individuals with congenital amusia, a disorder of musical and linguistic pitch processing. Future studies are required to investigate how and where along the auditory pathway acoustic features of speech/music sounds start to transform into internalized linguistic/musical percepts in the amusic brain, and how the amusic brainstem encodes more complex musical stimuli compared to normal controls.

## Conflict of Interest Statement

The authors declare that the research was conducted in the absence of any commercial or financial relationships that could be construed as a potential conflict of interest.
