# Supplementary material for: Brainstem encoding of speech and musical stimuli in congenital amusia: evidence from Cantonese speakers
Source: Front Hum Neurosci. 2015 Jan 6;8:1029. doi: 10.3389/fnhum.2014.01029 (PMC4297920; doi:10.3389/fnhum.2014.01029)
Supplement: Supplementary file 1 [file Table_1.PDF]

Table S1. Characteristics of the amusic (*n* = 14, all right-handed) and control (*n* = 14, all right-handed) groups. F = female; M = male; Age, education, and musical training are in years; scores on the six MBEA subtests (scale, contour, interval, rhythm, meter, and memory) are in number of correct responses out of 30 (Peretz et al., 2003); the pitch composite score is the sum of the scale, contour, and interval scores; MBEA global score is the percentage of correct responses out of the total 180 trials.

Subject	Group	Sex	Age	Education	Musical training	Scale	Contour	Interval	Rhythm	Meter	Memory	Pitch composite	MBEA global
A01	Amusic	F	21	16	0	24	22	22	22	20	28	68	76.67
A02	Amusic	M	20	14	0	26	16	20	21	23	25	62	72.78
A03	Amusic	F	21	15	0	23	23	18	22	20	28	64	74.44
A04	Amusic	F	47	13	2	26	19	19	22	19	24	64	71.67
A05	Amusic	F	43	15	0	22	26	24	17	19	27	72	75.00
A06	Amusic	F	21	18	2	21	21	19	27	20	25	61	73.89
A07	Amusic	M	18	12	0	21	24	20	22	25	30	65	78.89
A08	Amusic	F	19	13	0	16	22	18	20	12	19	56	59.44
A09	Amusic	F	18	12	1	21	20	17	24	19	29	58	72.22
A10	Amusic	F	18	12	0	21	22	21	25	23	27	64	77.22
A11	Amusic	F	22	16	0	23	22	20	25	27	29	65	81.11
A12	Amusic	M	22	16	0	22	21	22	25	25	26	65	78.33
A13	Amusic	F	48	11	4	20	22	18	23	16	27	60	70.00
A14	Amusic	M	19	14	6	21	22	22	26	28	30	65	82.78
C01	Control	F	22	17	0	26	28	29	30	30	29	83	95.56
C02	Control	F	19	14	0	29	28	25	24	29	30	82	91.67
C03	Control	F	24	17	4	27	25	27	30	28	30	79	92.78
C04	Control	F	21	16	2	29	28	29	29	30	30	86	97.22
C05	Control	F	22	16	0	30	27	29	29	30	28	86	96.11
C06	Control	M	22	17	3	28	24	29	30	30	28	81	93.89
C07	Control	F	42	18	0	28	25	27	30	26	30	80	92.22
C08	Control	F	45	23	3	28	27	25	29	19	29	80	87.22
C09	Control	F	21	14	0	30	29	28	29	28	29	87	96.11
C10	Control	F	41	18	1	24	30	25	28	24	29	79	88.89
C11	Control	M	19	16	6	28	30	30	30	30	30	88	98.89
C12	Control	M	18	12	0	30	27	29	30	28	29	86	96.11
C13	Control	M	21	15	0	27	29	27	30	30	30	83	96.11
C14	Control	M	19	13	0	27	29	27	30	27	28	83	93.33
