# Supplementary material for: Brainstem encoding of speech and musical stimuli in congenital amusia: evidence from Cantonese speakers
Source: Front Hum Neurosci. 2015 Jan 6;8:1029. doi: 10.3389/fnhum.2014.01029 (PMC4297920; doi:10.3389/fnhum.2014.01029)
Supplement: Supplementary file 2 [file Table_2.PDF]

Table S2. Results from the mixed-effects models on the effects of Tone (T1-6, F(5,130)), Group (amusic versus control, F(1,25)), Education (F(1,25)), and Tone × Group (F(5,130)) on FFR measures of speech in quiet. Significant effects are highlighted in boldface.

Effects		Tone						Group		Education	Tone × Group
		T1	T2	T3	T4	T5	T6	Amusic	Control		
Neural lag	Mean (SD)	8.88 (0.96)	8.79 (1.20)	8.57 (1.23)	8.41 (1.37)	8.85 (0.84)	8.73 (1.36)	8.66 (1.03)	8.75 (1.30)	-	-
	F	1.01						0.08		0.06	0.33
	p	0.418						0.781		0.814	0.893
Pitch strength	Mean (SD)	**0.60 (0.18)**	**0.72 (0.10)**	**0.75 (0.12)**	**0.74 (0.11)**	**0.76 (0.12)**	**0.75 (0.11)**	0.72 (0.11)	0.72 (0.16)	-	-
	F	**12.17**						0.41		**7.16**	0.37
	p	**< .001**						0.526		**0.013**	0.868
Pitch error	Mean (SD)	**4.87 (2.81)**	**9.09 (2.90)**	**3.36 (3.44)**	**2.77 (1.35)**	**3.06 (1.51)**	**3.37 (1.51)**	4.07 (2.74)	4.77 (3.63)	-	-
	F	**42.57**						1.42		2.94	0.55
	p	**< .001**						0.244		0.099	0.742
Stimulus-to-response correlation	Mean (SD)	**0.54 (0.29)**	**0.16 (0.33)**	**0.47 (0.46)**	**0.72 (0.25)**	**0.67 (0.22)**	**0.48 (0.32)**	0.53 (0.34)	0.48 (0.39)	-	-
	F	**10.46**						0.32		1.04	0.70
	p	**< .001**						0.574		0.319	0.627
Signal-to-noise ratio (SNR)	Mean (SD)	**6.97 (3.75)**	**10.20 (3.99)**	**9.65 (3.99)**	**8.91 (3.57)**	**8.76 (3.07)**	**8.15 (3.27)**	8.28 (3.02)	9.27 (4.26)	-	-
	F	**6.71**						0.85		3.27	0.77
	p	**< .001**						0.366		0.083	0.574
Root mean square (RMS) amplitude	Mean (SD)	**0.30 (0.05)**	**0.29 (0.05)**	**0.33 (0.05)**	**0.32 (0.05)**	**0.31 (0.04)**	**0.32 (0.05)**	0.30 (0.05)	0.32 (0.05)	-	-
	F	**3.98**						2.05		2.24	0.45
	p	**0.002**						0.164		0.147	0.813
F_0 (1st harmonic) amplitude	Mean (SD)	**-15.56 (3.29)**	**-17.07 (2.37)**	**-13.12 (3.47)**	**-15.01 (3.37)**	**-14.18 (2.86)**	**-14.78 (2.79)**	-15.21 (2.77)	-14.69 (3.65)	-	-
	F	**9.34**						0.43		4.19	1.22
	p	**< .001**						0.516		0.051	0.305
2nd harmonic amplitude	Mean (SD)	**-28.06 (2.98)**	**-26.49 (2.91)**	**-24.01 (4.79)**	**-22.53 (3.15)**	**-24.10 (3.03)**	**-24.23 (3.11)**	-24.48 (3.65)	-25.32 (3.94)	-	-
	F	**13.42**						1.06		0.75	0.85
	p	**< .001**						0.314		0.394	0.520
3rd harmonic amplitude	Mean (SD)	**-32.60 (4.53)**	**-32.59 (3.67)**	**-30.42 (4.61)**	**-30.57 (2.98)**	**-31.54 (3.14)**	**-33.10 (4.03)**	-31.12 (3.78)	-32.48 (4.03)	-	-
	F	**3.71**						1.94		1.35	0.51
	p	**0.004**						0.176		0.256	0.766

Effect of tone: Both groups demonstrated lower pitch strengths for Tone 1 than Tones 3-6 (all *p*s < 0.01). Both groups showed higher pitch errors for Tone 1 than Tones 4-5 (both *p*s < 0.05), and higher pitch errors for Tone 2 than the other five tones (all *p*s < 0.001). Both groups demonstrated lower stimulus-to-response correlations for Tone 2 than Tones 1 and 3-5 (all *p*s < 0.05), and higher stimulus-to-response correlations for Tone 4 than Tones 1-3. Both groups showed lower SNR for Tone 1 than Tone 2 ($p = 0.016$). RMS differed significantly across different tones for both groups, although none of the pairwise comparisons reached significance in post-hoc analysis. Both groups showed larger 1st harmonic amplitudes for Tone 3 than Tone 1 and Tone 2 (both *p*s < 0.05), and for Tone 5 than Tone 2 ($p = 0.007$). Both groups showed smaller 2nd harmonic amplitudes for Tone 1

than Tones 3-6 (all *p*s < 0.001), and for Tone 2 than Tone 4 ($p < 0.001$). 3rd harmonic amplitudes differed significantly across different tones for both groups, although none of the pairwise comparisons reached significance in post-hoc analysis.

Effect of education: Years of education showed a negative impact on pitch strength: the more education participants received, the lower their pitch strengths.
