# Supplementary material for: Brainstem encoding of speech and musical stimuli in congenital amusia: evidence from Cantonese speakers
Source: Front Hum Neurosci. 2015 Jan 6;8:1029. doi: 10.3389/fnhum.2014.01029 (PMC4297920; doi:10.3389/fnhum.2014.01029)
Supplement: Supplementary file 3 [file Table_3.PDF]

Table S3. Results from the mixed-effects models on the effects of Tone (Tone 1 versus Tone 6, F(1,26)), Group (Amusic versus Control, F(1,25)), noise (0 dB SNR versus 20 dB SNR, F(1,52)), Education (F(1,25)), Tone × Group (F(1,26)), Tone × Noise (F(1,52)), Group × Noise (F(1,52)), Tone × Group × Noise (F(1,52)) on FFR measures of speech in noise. Significant effects are highlighted in boldface.

Effects		Tone		Group		Noise		Education	Tone × Group	Tone × Noise	Group × Noise	Tone × Group × Noise
		T1	T6	A	C	0 dB	20 dB					
Neural lag	Mean (SD)	8.80 (1.03)	8.76 (0.86)	8.63 (0.59)	8.92 (1.19)	**8.59 (0.83)**	**8.96 (1.02)**	-	-	-	-	-
	F	0.03		1.59		**23.09**		0.00	**4.65**	0.32	1.11	1.42
	p	0.858		0.219		**< .001**		0.997	**0.040**	0.573	0.297	0.239
Pitch strength	Mean (SD)	**0.58 (0.14)**	**0.66 (0.11)**	0.60 (0.14)	0.64 (0.12)	**0.59 (0.13)**	**0.65 (0.13)**	-	-	-	-	-
	F	**16.26**		2.43		**13.68**		**11.57**	0.22	0.04	3.68	0.15
	p	**< .001**		0.132		**< .001**		**0.002**	0.644	0.849	0.061	0.701
Pitch error	Mean (SD)	**4.77 (2.44)**	**3.62 (1.25)**	4.34 (1.93)	4.05 (2.11)	**4.62 (1.97)**	**3.77 (1.98)**	-	-	-	-	-
	F	**9.79**		0.34		**15.79**		**6.08**	0.14	0.54	**7.19**	2.02
	p	**0.004**		0.562		**< .001**		**0.021**	0.712	0.467	**0.010**	0.161
Stimulus-to-response correlation	Mean (SD)	0.50 (0.32)	0.46 (0.25)	0.49 (0.29)	0.46 (0.28)	0.47 (0.31)	0.49 (0.26)	-	-	-	-	-
	F	0.89		0.50		0.11		**7.80**	0.03	1.74	2.28	0.43
	p	0.354		0.488		0.747		**0.010**	0.856	0.193	0.137	0.515
Signal-to-noise ratio (SNR)	Mean (SD)	**5.44 (2.39)**	**6.31 (2.64)**	5.41 (2.49)	6.34 (2.53)	**5.11 (2.43)**	**6.64 (2.45)**	-	-	-	-	-
	F	**6.75**		2.28		**22.74**		**9.63**	0.27	0.02	0.40	0.55
	p	**0.015**		0.143		**< .001**		**0.005**	0.607	0.878	0.529	0.461
Root mean square (RMS) amplitude	Mean (SD)	0.27 (0.04)	0.27 (0.04)	0.26 (0.04)	0.28 (0.04)	**0.26 (0.04)**	**0.28 (0.04)**	-	-	-	-	-
	F	0.00		3.22		**6.45**		1.50	1.23	0.01	0.01	0.00
	p	0.960		0.085		**0.014**		0.233	0.277	0.910	0.943	0.973
F_0 (1st harmonic) amplitude	Mean (SD)	-16.31 (3.45)	-16.43 (2.76)	**-17.07 (3.35)**	**-15.66 (2.71)**	**-17.06 (3.51)**	**-15.67 (2.50)**	-	-	-	-	-
	F	0.05		**5.11**		**6.88**		**6.43**	0.04	0.41	1.15	0.02
	p	0.817		**0.033**		**0.011**		**0.018**	0.835	0.524	0.288	0.879
2nd harmonic amplitude	Mean (SD)	**-27.01 (3.12)**	**-24.67 (2.80)**	-25.64 (2.96)	-26.04 (3.39)	-25.60 (3.42)	-26.07 (2.92)	-	-	-	-	-
	F	**13.02**		0.29		1.45		0.00	0.39	2.39	1.28	0.63
	p	**0.001**		0.598		0.234		0.979	0.540	0.128	0.263	0.431
3rd harmonic amplitude	Mean (SD)	-32.04 (3.59)	-33.07 (3.18)	-32.55 (3.51)	-32.56 (3.35)	-32.55 (3.50)	-32.56 (3.36)	-	-	-	-	-
	F	1.98		0.00		0.00		0.40	0.09	0.71	0.68	0.37
	p	0.171		0.993		0.974		0.535	0.763	0.403	0.413	0.547

Effect of tone: Both groups showed higher pitch strengths, smaller pitch errors, bigger signal-to-noise ratios, and larger 2nd harmonic amplitudes in FFRs to Tone 6 in noise than to Tone 1 in noise.

Effect of group: Controls showed larger 1st harmonic amplitudes in FFRs to speech in noise than amusics.

Effect of noise: Both groups showed longer neural lags, higher pitch strengths, smaller pitch errors, bigger signal-to-noise ratios, bigger root mean square amplitudes, and larger 1st harmonic amplitudes in FFRs to tones under 20 dB SNR than to tones under 0 dB SNR.

Effect of education: Years of education showed a negative impact on pitch strength, pitch error, stimulus-to-response correlation, signal-to-noise ratio, and 1st harmonic amplitude: the more years of education participants received, the worse those FFR measures.

Tone × Group interaction on neural lag: Controls showed longer neural lags than amusics for Tone 1 in noise, $t(54) = -3.09$, $p = 0.003$, control mean (SD): 9.19 (1.29), amusic mean (SD): 8.40 (0.41), but not for Tone 6 in noise, $t(54) = 0.90$, $p = 0.370$, control mean (SD): 8.65 (1.02), amusic mean (SD): 8.86 (0.66).

Group × Noise interaction on pitch error: Controls showed less pitch errors than amusics when SNR = 0 dB, $t(54) = 1.67$, $p = 0.101$, control mean (SD): 4.19 (1.74), amusic mean (SD): 5.05 (2.12), but more pitch errors when SNR = 20 dB, $t(54) = -0.53$, $p = 0.597$, control mean (SD): 3.91 (2.44), amusic mean (SD): 3.63 (1.42).
