# Supplementary material for: Brainstem encoding of speech and musical stimuli in congenital amusia: evidence from Cantonese speakers
Source: Front Hum Neurosci. 2015 Jan 6;8:1029. doi: 10.3389/fnhum.2014.01029 (PMC4297920; doi:10.3389/fnhum.2014.01029)
Supplement: Supplementary file 4 [file Table_4.PDF]

Table S4. Results from the mixed-effects models on the effects of Tone (high = 150 Hz versus low = 112 Hz, F(1,26)), Group (amusic versus control, F(1,25)), Education (F(1,25)), and Tone × Group (F(1,26)) on FFR measures of cello tones. Significant effects are highlighted in boldface.

Effects		Tone		Group		Education	Tone × Group
		High	Low	Amusic	Control		
Neural lag	Mean (SD)	10.40 (1.65)	10.61 (1.65)	10.33 (1.39)	10.69 (1.85)	-	-
	F	0.37		0.49		1.90	0.81
	p	0.549		0.493		0.180	0.378
Pitch strength	Mean (SD)	**0.35 (0.13)**	**0.52 (0.14)**	0.45 (0.16)	0.42 (0.15)	-	-
	F	**37.55**		0.18		0.16	0.17
	p	**< 0.001**		0.672		0.696	0.686
Pitch error	Mean (SD)	**12.91 (5.73)**	**8.37 (4.79)**	10.51 (6.74)	10.77 (4.58)	-	-
	F	**14.46**		0.03		0.15	1.56
	p	**< 0.001**		0.874		0.704	0.223
Stimulus-to-response correlation	Mean (SD)	**-0.01 (0.12)**	**0.36 (0.40)**	0.17 (0.38)	0.18 (0.32)	-	-
	F	**21.21**		0.01		0.62	0.66
	p	**< 0.001**		0.931		0.440	0.425
Signal-to-noise ratio (SNR)	Mean (SD)	3.94 (1.82)	4.87 (2.93)	4.38 (2.15)	4.45 (2.77)	-	-
	F	3.48		0.01		1.06	0.02
	p	0.073		0.931		0.313	0.878
Root mean square (RMS) amplitude	Mean (SD)	0.24 (0.03)	0.26 (0.04)	0.25 (0.03)	0.25 (0.04)	-	-
	F	1.82		0.01		0.23	0.80
	p	0.189		0.947		0.636	0.380
F_0 (1st harmonic) amplitude	Mean (SD)	**-21.72 (4.54)**	**-19.25 (4.46)**	-20.38 (5.26)	-20.59 (4.01)	-	-
	F	**5.89**		0.02		0.03	0.12
	p	**0.022**		0.883		0.861	0.733
2nd harmonic amplitude	Mean (SD)	**-32.06 (3.09)**	**-29.97 (1.94)**	-31.58 (3.01)	-30.44 (2.43)	-	-
	F	**9.34**		2.77		0.29	0.60
	p	**0.005**		0.109		0.592	0.444
3rd harmonic amplitude	Mean (SD)	**-35.73 (2.39)**	**-32.88 (2.85)**	-34.01 (2.45)	-34.60 (3.44)	-	-
	F	**17.31**		0.66		1.40	0.04
	p	**< 0.001**		0.426		0.249	0.845

Effect of tone: Both groups showed higher pitch strengths, smaller pitch errors, bigger stimulus-to-response correlations, and larger 1st, 2nd, and 3rd harmonic amplitudes in FFRs to the low cello tone (112 Hz) than to the high cello tone (150 Hz).
