# Supplementary material for: Brainstem encoding of speech and musical stimuli in congenital amusia: evidence from Cantonese speakers
Source: Front Hum Neurosci. 2015 Jan 6;8:1029. doi: 10.3389/fnhum.2014.01029 (PMC4297920; doi:10.3389/fnhum.2014.01029)

Figure S1. Grand average waveforms of FFRs (from two participants) to the six Cantonese lexical tones with the earphone removed from the ear canal. As can be seen, with no sound input routed to the ear, the waveforms only represent random noise with a very low amplitude and no pitch, suggesting that our FFR recordings were not affected by any EM or transducer artifacts.

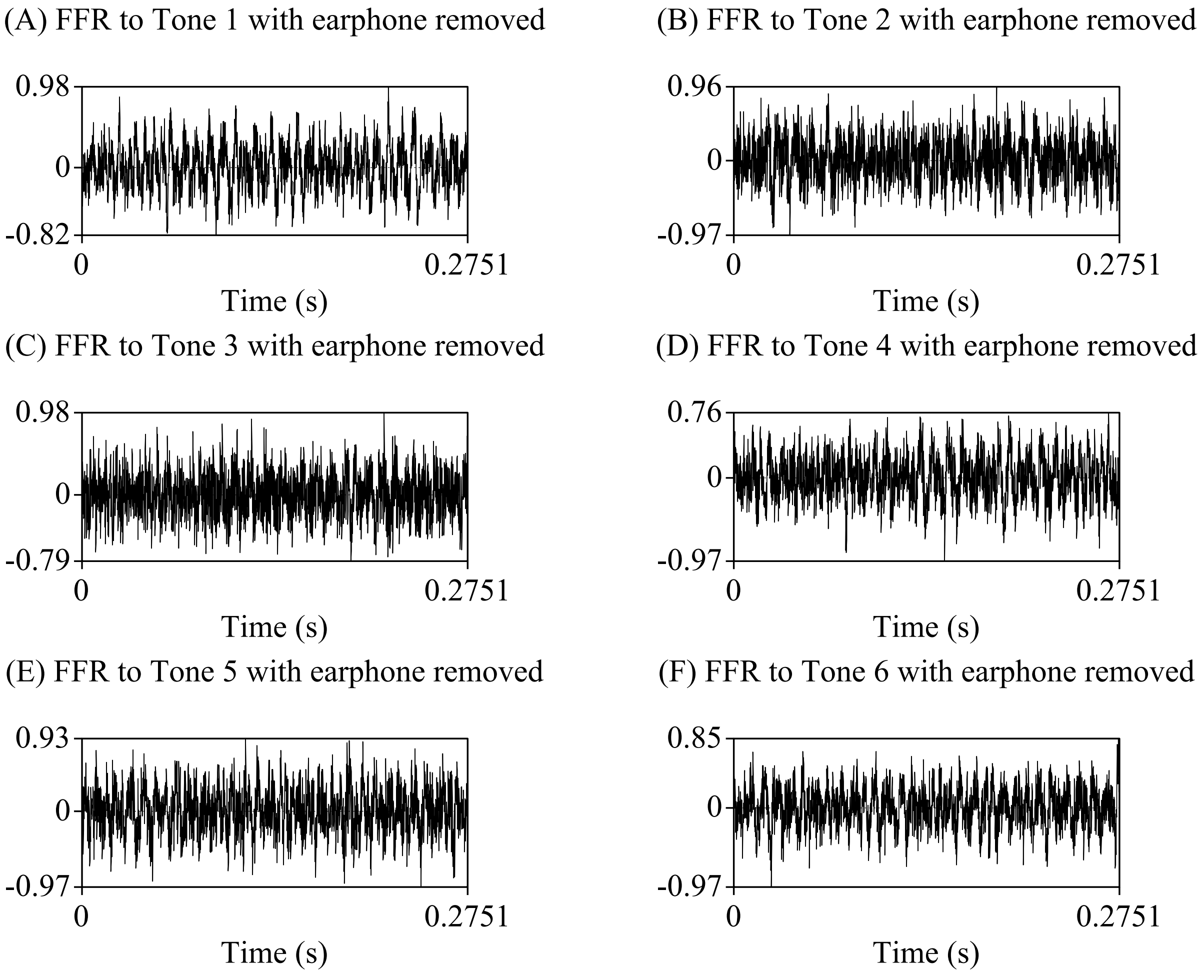

Supplement: Supplementary file 5 [file Image_1.PDF]
